# Large-scale genome-wide association study to identify causal relationships and potential mediators between education and autoimmune diseases

**DOI:** 10.3389/fimmu.2023.1249017

**Published:** 2023-12-07

**Authors:** Yingjie Li, Jingwei Zhang, Jie Wen, Mingren Liu, Wanyao Liu, Yongzhen Li

**Affiliations:** ^1^ Department of Infectious Diseases, The Second Xiangya Hospital, Central South University, Changsha, China; ^2^ The Institution of Hepatology, Central South University, Changsha, China; ^3^ Department of Neurosurgery, Xiangya Hospital, Central South University, Changsha, China; ^4^ Hypothalamic Pituitary Research Center, Xiangya Hospital, Central South University, Changsha, China; ^5^ National Clinical Research Center for Geriatric Disorders, Xiangya Hospital, Central South University, Changsha, China; ^6^ Xiangya School of Medicine, Central South University, Changsha, China; ^7^ Department of Pediatrics, The Second Xiangya Hospital, Central South University, Changsha, China

**Keywords:** Mendelian randomization, education, autoimmune diseases, causal relationship, Transcriptome-wide association study

## Abstract

**Objectives:**

Epidemiological studies suggested a potential connection between education and autoimmune disorders. This study investigated the possible cause-and-effect relationship using a Mendelian randomization approach.

**Methods:**

We explored the causality between four education traits (n = 257,841~1,131,881) and 22 autoimmune diseases. The mediating role of smoking (632,802 individuals), BMI (681,275 individuals), alcohol (335,394 individuals), and income (397,751 individuals) was also investigated. Transcriptome-wide association study (TWAS) and enriched signaling pathways analysis were used to investigate the underlying biological mechanisms.

**Results:**

Especially, higher cognitive performance was protective for psoriasis (odds ratio (OR) = 0.69, 95% confidence interval (CI) = 0.60-0.79, *p* = 6.12×10^-8^), rheumatoid arthritis (RA) (OR = 0.75, 95% CI = 0.67-0.83, *p* = 4.62×10^-6^), and hypothyroidism (OR = 0.83, 95% CI = 0.77-0.90, *p* = 9.82×10^-6^). Higher levels of educational attainment decreased risks of psoriasis (OR = 0.61, 95% CI = 0.52-0.72, *p* = 1.12×10^-9^), RA (OR = 0.68, 95% CI = 0.59-0.79, *p* = 1.56×10^-7^), and hypothyroidism (OR = 0.80, 95% CI = 0.72-0.88, *p* = 5.00×10^-6^). The completion of highest-level math class genetically downregulates the incidence of psoriasis (OR = 0.66, 95% CI = 0.58-0.76, *p* = 2.47×10^-9^), RA (OR = 0.71, 95% CI = 0.63-0.81, *p* = 5.28×10^-8^), and hypothyroidism (OR = 0.85, 95% CI = 0.79-0.92, *p* = 8.88×10^-5^). Higher self-reported math ability showed protective effects on Crohn’s disease (CD) (OR = 0.67, 95% CI = 0.55-0.81, *p* = 4.96×10^-5^), RA (OR = 0.76, 95% CI = 0.67-0.87, *p* = 5.21×10^-5^), and psoriasis (OR = 0.76, 95% CI = 0.65-0.88, *p* = 4.08×10^-4^). Protein modification and localization, response to arsenic-containing substances may participate in the genetic association of cognitive performance on UC, RA, psoriasis, and hypothyroidism. According to mediation analyses, BMI, smoking, and income served as significant mediators in the causal connection between educational traits and autoimmune diseases.

**Conclusion:**

Higher levels of education-related factors have a protective effect on the risk of several autoimmune disorders. Reducing smoking and BMI and promoting income equality can mitigate health risks associated with low education levels.

## Introduction

Autoimmune diseases are a breach of immune tolerance that cannot differentiate self from non-self, affect 3-5% of the population, and have more than 100 different types (1). Some have lesions that are localized to specific organs, such as primary sclerosing cholangitis (PSC), while others involve multiple organs and systems, such as systemic lupus erythematosus (SLE) (2). The exact cause of autoimmune diseases remains uncertain and may be influenced by a combination of genetic factors and epigenetic changes caused by environmental factors (3), lifestyle (4), and intestinal microbiota (5). However, it’s worth noting that observational studies don’t always prove causality because of confounding factors and reverse causal effects. More advanced tools are required to investigate the potential etiologies of autoimmune disorders.

Mendelian Randomization (MR) is a research method that enables the investigation of the causal relationship between the factors of interest and outcomes using instrumental variables (IVs) (6). When performing an MR study, genetic variants highly linked to exposure and fulfill specific criteria are utilized as instrumental variables (IVs) to probe the cause-and-effect relationship with an outcome. Proper execution of MR can help mitigate bias caused by confounding environmental factors, as these variants are randomly allocated during conception. Thus, the design of MR closely resembles that of a randomized controlled trial (RCT). When selecting exposures and outcomes, the sample size is crucial as it can lead to weak instrumental variable bias (7).

One of the main focus areas in the summary level of GWAS data is education, which includes cognitive ability, educational achievement, highest-level math class taken, and self-reported math proficiency. The risk factors associated with education, including biological (such as BMI), behavioral (such as smoking and alcohol use), and psychosocial factors (such as income), have been extensively studied in relation to cardiovascular disease (8, 9) psychiatric disorders (10), and neurodegenerative diseases (11, 12). Some observational studies also suggested that higher education has a protective effect on psoriasis (13), RA (14), and SLE (15).

To obtain the causal relationship between education on autoimmune diseases and potential mediator factors, we applied a two-sample and a two-step MR using cognitive ability, educational achievement, highest-level math class taken, and self-reported math proficiency as exposures, 22 traits of autoimmune disorders [asthma and allergy (AA), systemic lupus erythematosus (SLE), rheumatoid arthritis (RA), psoriasis (PsO), hypothyroidism, hyperthyroidism, interstitial lung disease (ILD) related to systemic autoimmune disease, other autoimmune hemolytic anemias (AIHA), scleroderma, sicca syndrome (SS), ankylosing spondylitis (AS), amyotrophic lateral sclerosis (ALS), asthma, Crohn’s disease (CD), ulcerative colitis (UC), celiac disease (CeD), irritable bowel syndrome (IBS), multiple sclerosis (MS), primary biliary cholangitis (PBC), primary sclerosing cholangitis (PSC), and type 1 diabetes (T1D)] as outcomes, and BMI, alcohol use, and income as potential mediators. We also performed TWAS and GO: BP enrichment analyses to explore potential transcriptomic and biological process basis for the genetic associations.

## Materials and methods

### Mendelian randomization

To conduct an MR study, the genetic variants (single nucleotide polymorphisms, SNPs) must meet several essential requirements to be used as IVs. Firstly, they should be strongly connected with the exposure of being studied (relevance assumption). Secondly, they are not associated with confounders of the risk factor-outcome association (independence assumption). Finally, it is crucial that the SNPs only impact the outcomes through the exposures (restriction assumption) (16).

#### Data sources for exposures

The available GWAS summary statistics correlated with cognitive performance (n= 257,841), educational attainment (n= 1,131,881), highest-level math class completion (n= 430,445), and self-reported math ability (n= 564,698) was derived from the GWAS meta-analysis of European ancestry of the Social Science Genetic Association Consortium (SSGAC) (17). This MR study used a meta-analysis of cognitive performance based on published data from the Cognitive Genomics Consortium (COGENT) and new analyses of the United Kingdom Biobank (UKB) of European ancestry (18). The study collected data on educational attainment from 766,345 participants of European ancestry who were over 30 years old, and one standard deviation represents an increase of 4.2 years of education completion (17). Detailed information of data sources for exposures is in **Supplementary Table 1**.

#### Data sources for outcomes

The GWAS data of 22 traits of autoimmune diseases including asthma and allergy (AA) (n= 197,963), systemic lupus erythematosus (SLE) (n= 257,998), rheumatoid arthritis (RA) (n= 232,501), psoriasis (n= 339,050), hypothyroidism (n= 287,247), hyperthyroidism (n= 257,552), interstitial lung disease (ILD) related to systemic autoimmune disease (n= 341,986), other autoimmune hemolytic anemias (AIHA) (n= 341,986), scleroderma (n= 322,208), sicca syndrome (SS) (n= 334,362), ankylosing spondylitis (AS) (n= 251,394), and autoimmune diseases (n= 342,499) are from the FINNGEN dataset (19), amyotrophic lateral sclerosis (ALS) (n= 138,086) (20), asthma (n= 408,422) (21), Crohn’s disease (CD) (n= 40,266) (22), celiac disease (CeD) (n= 456,348) (23), irritable bowel syndrome (IBS) (n= 486,601), multiple sclerosis (MS) (n= 115,803) (24), primary biliary cholangitis (PBC) (n= 24,510) (25), primary sclerosing cholangitis (PSC) (n= 14,890) (26), type 1 diabetes (T1D) (n= 24,840) (27), ulcerative colitis (UC) (n= 45,975) (26). Detailed information of data sources for outcomes is in **Supplementary Table 1**.

#### Data sources for mediators

The summary-level GWAS data correlated with smoking (n= 632,802) and alcohol use (n= 335,394) were derived from a meta-analysis of European participants conducted by Mengzhen Liu et al. (28). The summary-level GWAS data correlated with BMI (n= 681,275, each SD =4.8 kg/m^2^) was gathered through a meta-analysis of individuals from Europe conducted by Loic Yengo et al. (29) The GWAS data related to average total household income before tax (n= 397,751; GWAS ID: ukb-b-7408) was derived from the UK Biobank (http://www.nealelab.is/uk-biobank/) (30) of European ancestry, conducted through an open-ended questionnaire. Detailed information of data sources for mediators is in **Supplementary Table 1**.

#### Instrumental variable selection and data harmonization

To choose the appropriate genetic tools for each of the four exposures (including cognitive performance, educational attainment, highest-level math class completed, and self-reported math ability), we used the default settings in the R package TwoSampleMR (31, 32) to identify significant genetic variations, we extracted SNPs with a P-value less than 5.0×10^-8^. We used standard clumping criteria to identify distinct SNPs. Specifically, we set the clumping window to 10,000 kb and applied an LD r^2^ threshold of 0.001. We then calculated the R^2^ and F-statistics to determine the strength of the identified genetic variations in explaining the proportion of exposure variance (33). $(F=\frac{R^{2}/K}{\left(1-R^{2}\right)/\left(N-K-1\right)}$ , K= n SNPs and N= n sample, the F-number should be greater than 10). Detailed information on instrumental SNPs for exposures and mediators is in **Supplementary Tables 2–9**.

#### Two-sample Mendelian randomization analyses

To account for variant heterogeneity and the pleiotropy effect, we employed three distinct techniques in MR analysis: Random-effect inverse-variance weighted (IVW), MR Egger, and weighted median (32). We also removed any SNPs related to 22 traits of autoimmune diseases mentioned above and outliers identified with MRPRESSO. We then utilized MR-Egger and weighted median in conjunction with the primary outcome of IVW. These approaches are recognized for producing more reliable results in various situations, albeit with slightly broader confidence intervals. MR-Egger considers the possibility of pleiotropic effects in all genetic variants, but it is essential that these effects do not influence the variant-exposure association (34). To conduct a test on a global scale, a significant two-sided P-value of 0.05 was established. For regional-level analyses utilizing 88 MR estimates, a Bonferroni-corrected P-value of 0.05/88 (5.68×10^-4^) was used, while any p<0.05 was set as nominally significant.

#### Two-step Mendelian randomization analyses

To investigate the extent to which education affects autoimmune diseases via mediators (including BMI, income, smoking, and alcohol use), a 2-step MR by the product of the coefficient method was performed using public GWAS summary statistics (35, 36). The first step involved estimating the impact between education and BMI, alcohol consumption, smoking habits, and income. Following this, we investigated the genetic predisposition of these mediators on autoimmune disorders. The overall effect of education was divided into a direct effect (the genetic predisposition between education and autoimmune disorders independent of the mediator) and an indirect effect (the genetic predisposition between education and autoimmune disorders by the mediator). The mediating effect from the exposure to the outcome was calculated using the product of coefficients method, which involved converting ORs for binary outcomes to log ORs. We then analyzed the data and calculated the proportion of the effect. Standard errors were calculated by the Delta method.

### Transcriptome-wide association study and enriched signaling pathways analysis

**Figure 1:**
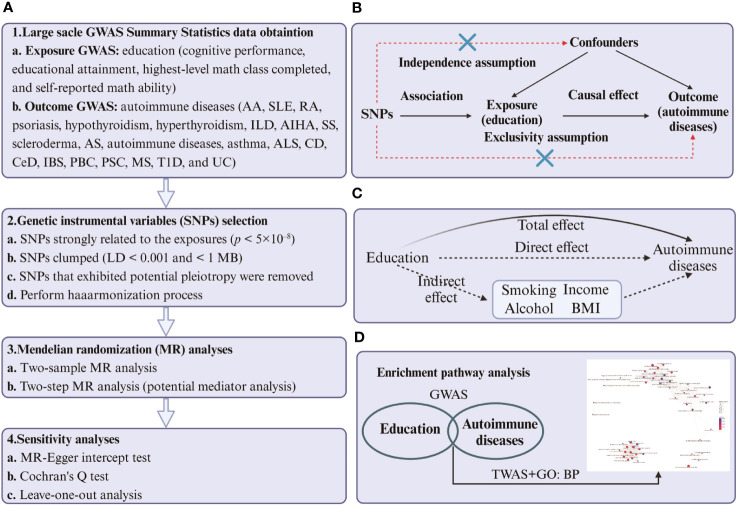
**(A)** Four steps of two-sample and two-step MR analysis of cause-effect of education-related factors on autoimmune diseases. The first step is to obtain the summary-level GWAS statistics of exposures, outcomes, and mediators. The second step is to select the qualified instrumental variables (SNPs), the third step is to perform the two-sample and two-step MR, and the last step is to conduct sensitivity analysis. **(B)** Design of the two-sample Mendelian randomization study. Three core assumptions were as follows: 1) the SNPs should be strongly associated with education-related factors; 2) the SNPs should not be related to confounders; 3) the SNPs should not be directly associated with autoimmune diseases. **(C)** The total effect (β_0_) was decomposed into the cause-effect of education-related factors on mediators (β_1_) and the cause-effect of mediators on autoimmune diseases (β_2_). The indirect effect equals β_0_-β_1_×β_2_. **(D)** The process of TWAS and GO: BP enrichment analyses. The first step is to identify the common genes between education and autoimmune diseases. The second step was to perform an enrichment analysis of biological processes targeting the transcriptome corresponding to the genes. The figure was built by BioRender.

We used the FUSION (37) method to transform GWAS into TWAS. This was done by utilizing a linear model of expression quantitative trait loci (eQTL) to estimate gene expression from European Blood RNA-seq Genotype-Tissue Expression version 8 (GTEx v8) (38). We then used the TWAS method to identify homozygous gene clusters linked to education measures and autoimmune disease traits. After analyzing the genes involved, we proceeded to conduct GO enrichment analyses (39) to better comprehend the biological processes that link education and autoimmune diseases. We used R packages such as clusterProfiler (40), enrichplot, and DOSE (41) to perform the analyses based on the Gene Ontology database (42, 43). The P-value was calculated using Fisher’s Combined P-value (FCP) method. As we only obtained cognitive performance data from the exposure, we performed TWAS and GO:BP enrichment analyses solely on positive cognitive performance results. The experimental design flow chart of MR and TWAS is shown in **Figure 1**.

### Sensitivity analyses

To obtain accurate estimates, we thoroughly evaluated horizontal pleiotropy through MR-Egger intercept testing and leave-one-out analyses. We also utilized Cochran’s Q tests to detect heterogeneity (44) and funnel plots to assess the possible presence of pleiotropy.

### Ethics approval

The study solely relied upon publicly available information, resulting in the waiver of ethical approval. Each of the studies that contributed to the GWAS has information on ethical approval and participant consent in their original publications. No specific ethical approval is required in this study.

## Results

### Effects of education on autoimmune diseases

**Figure 2:**
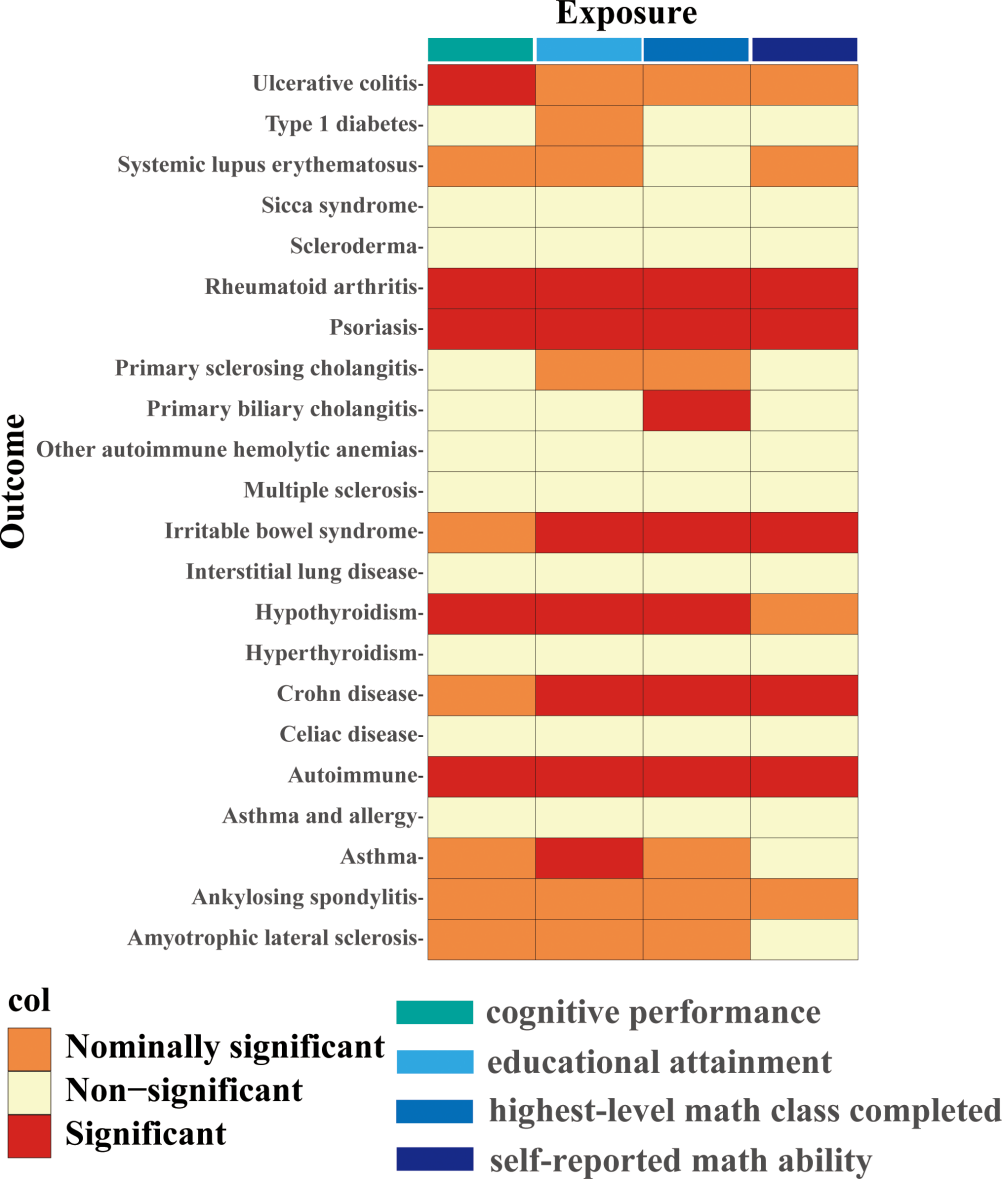
IVW estimates from cognitive performance, educational attainment, highest-level math class completed, and self-reported math ability on 22 traits of autoimmune diseases. The color of each block represents the IVW-derived P-values of every MR analysis. P-values of< 0.05 were shown in orange and set as nominal significant, and P-values of > 0.05 were shown in yellow and set as non-significant. P value< 5.68×10^-4^ is set as significant and shown in red.

Four different measures of education (cognitive performance, educational attainment, highest-level math class completed, and self-reported math ability) affect different autoimmune diseases to varying degrees. The complete results of the inverse variance-weighted (IVW) method are shown in **Figure 2**.

**Figure 3:**
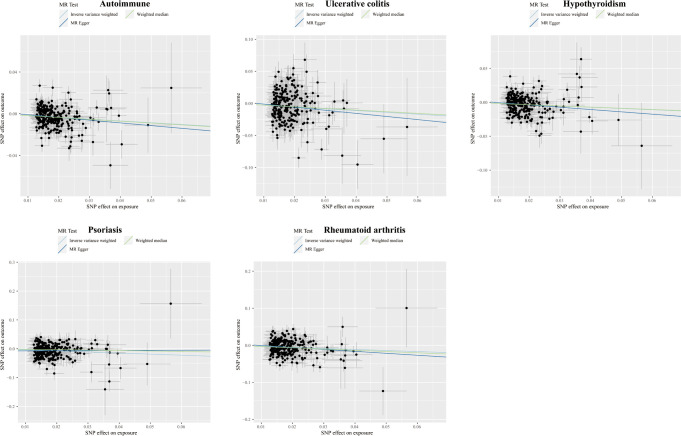
Scatter plots displaying the significant outcomes of MR effect regarding the impact of cognitive performance on autoimmune disorders. The x-axis represents the genetic association with cognitive performance risk; the y-axis represents the genetic association with the risk of autoimmune diseases, UC, hypothyroidism, psoriasis, and RA.

**Figure 4:**
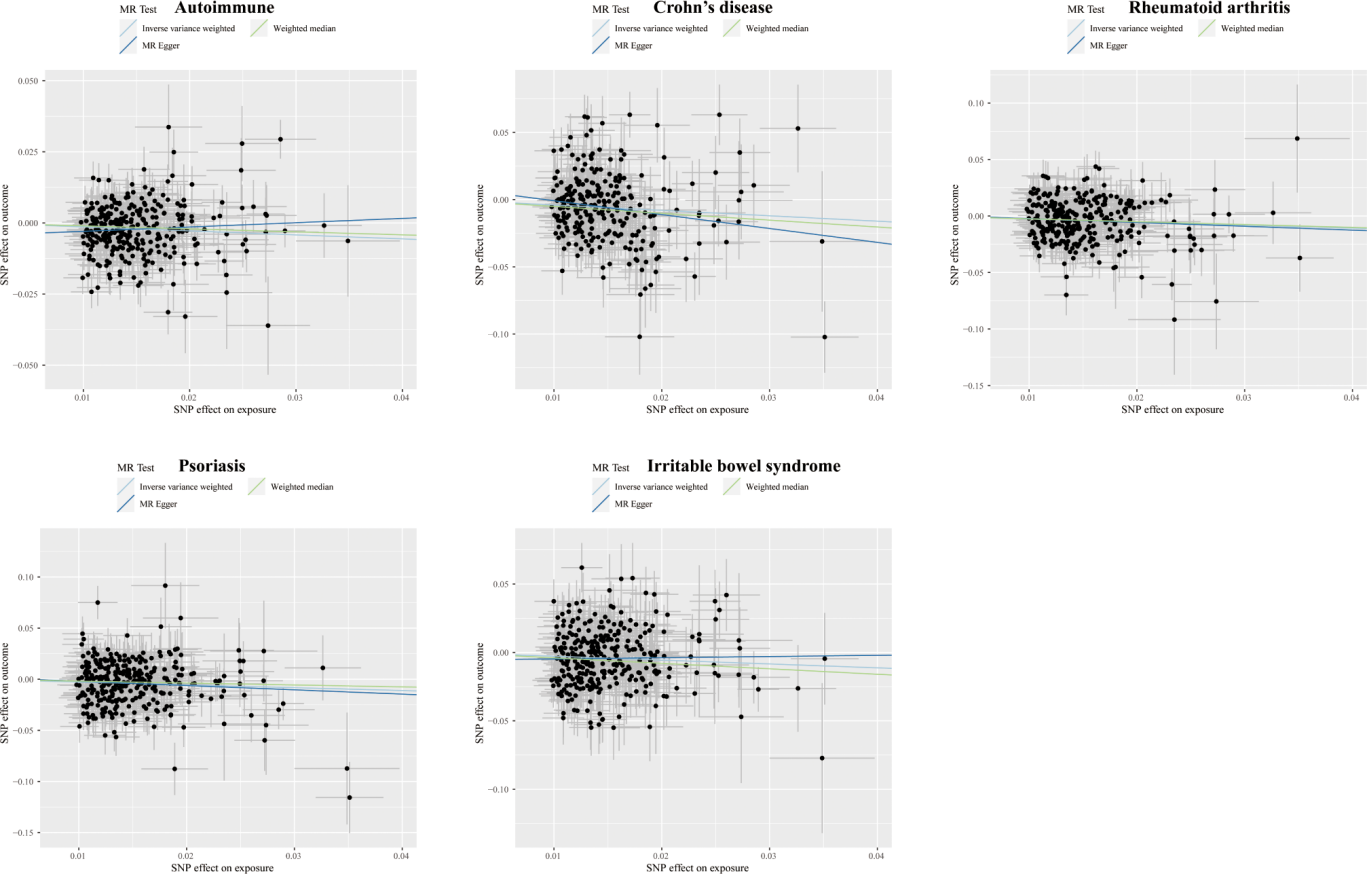
Scatter plots displaying the significant outcomes of MR effect regarding the impact of self-reported ability on autoimmune disorders. The x-axis represents the genetic association with self-reported ability risk; the y-axis represents the genetic association with the risk of autoimmune diseases, CD, RA, psoriasis, and IBS.

**Figure 5:**
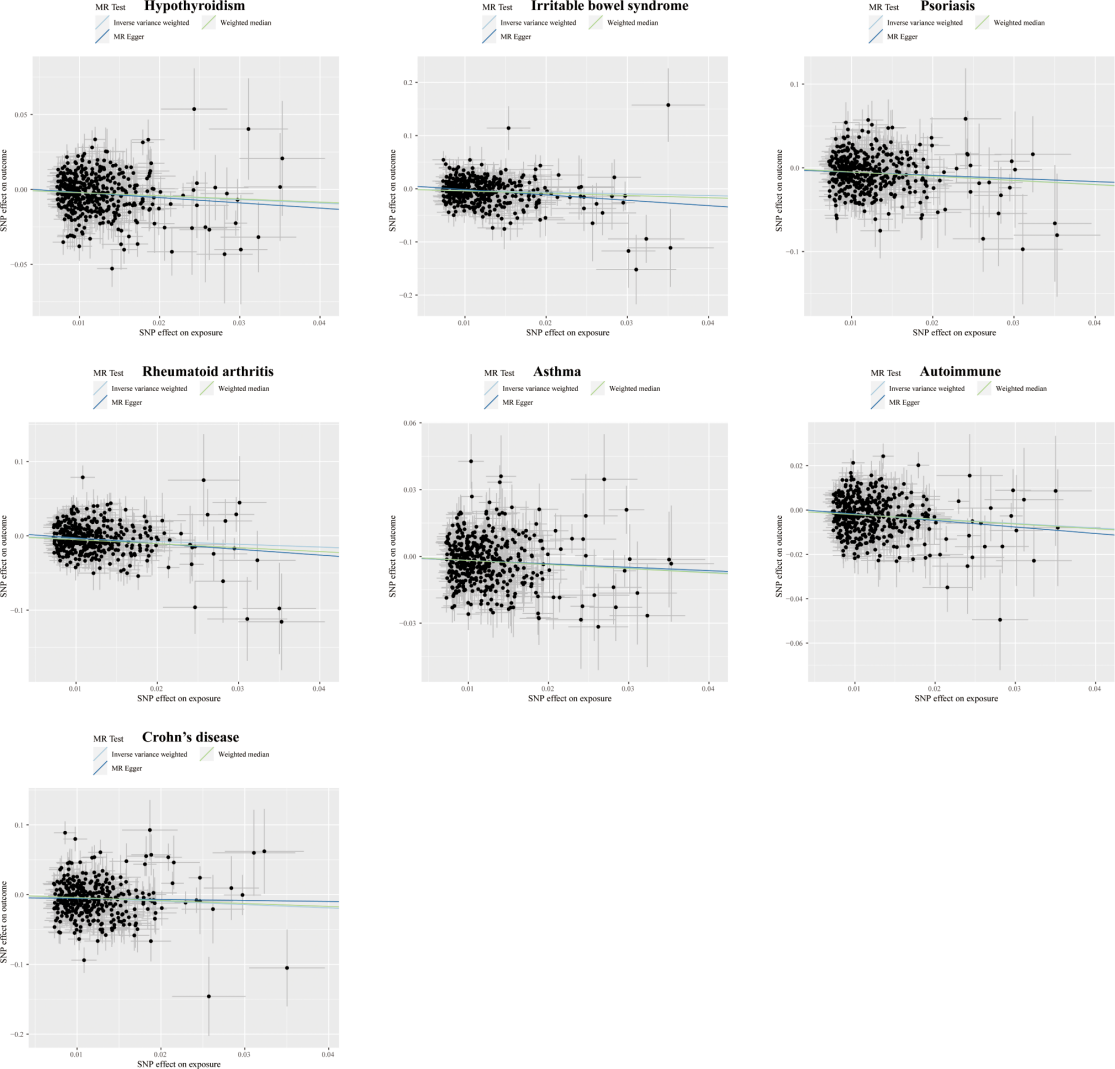
Scatter plots displaying the significant outcomes of the MR effect regarding the impact of educational attainment on autoimmune disorders. The x-axis represents the genetic association with educational attainment risk; the y-axis represents the genetic association with the risk of hypothyroidism, IBS, psoriasis, RA, asthma, autoimmune diseases, and CD.

**Figure 6:**
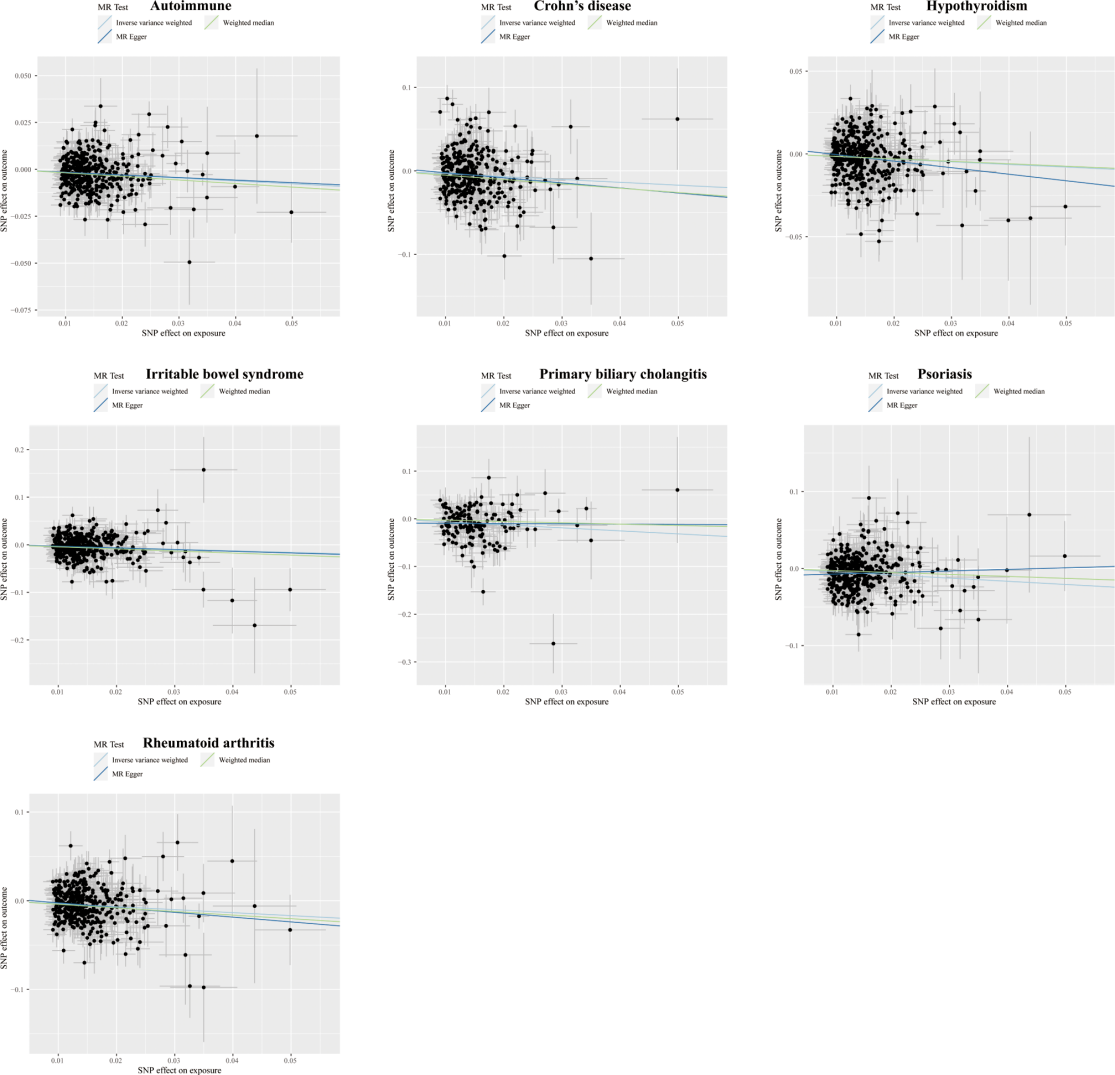
Scatter plots displaying the significant outcomes of MR effect regarding the impact of the completion of highest-level math class on autoimmune disorders. The x-axis represents the genetic association with the completion of the highest-level math class risk; the y-axis represents the genetic association with the risk of autoimmune diseases, CD, hypothyroidism, IBS, PBC, psoriasis, and RA.

Higher cognitive performance can downregulate risks of autoimmune disorders, including autoimmune diseases, psoriasis, RA, hypothyroidism, and UC, with the OR of 95% CI of 0.84 (0.80, 0.90), 0.69 (0.60, 0.79), 0.75 (0.67, 0.83), 0.83 (0.77, 0.90), and 0.76 (0.64, 0.88), respectively. The P-values of each trait were 1.06×10^-9^, 6.12×10^-8^, 4.62×10^-6^, 9.82×10^-6^, and 4.72×10^-4^, respectively; an increase of completion of 4.2 years of education was protective for autoimmune diseases, psoriasis, RA, hypothyroidism, asthma, CD, and IBS, and the ORs with 95% CI were 0.82 (0.76, 0.87), 0.61 (0.52, 0.72), 0.68 (0.59, 0.79), 0.80 (0.72, 0.88), 0.84 (0.77, 0.90), 0.62 (0.50, 0.77), and 0.73 (0.62, 0.85) with the P-values for each disease of 1.44×10^-9^, 1.12×10^-9^, 1.56×10^-7^, 5.00×10^-6^, 6.63×10^-6^, 1.28×10^-5^, and 9.95×10^-5^, respectively; highest-level math class completed is related to decreased incidence of autoimmune diseases, psoriasis, RA, IBS, hypothyroidism, PBC, and CD. The ORs with 95% CI were 0.86 (0.81, 0.91), 0.66 (0.58, 0.76), 0.71 (0.63, 0.81), 0.73 (0.63, 0.83), 0.85 (0.79, 0.92), 0.53 (0.38, 0.74), and 0.71 (0.59, 0.85), with P-values of 1.05×10^-7^, 2.47×10^-9^, 5.28×10^-8^, 5.25×10^-6^, 8.88×10^-5^, 1.54×10^-4^, and 2.31×10^-4^, respectively. Higher self-reported math ability shows protective effects against autoimmune diseases, CD, RA, IBS, and psoriasis. The OR with 95% CI for each trait was 0.87 (0.82, 0.92), 0.67 (0.55, 0.81), 0.76 (0.67, 0.87), 0.75 (0.65, 0.88), 0.76 (0.65, 0.88) with P-values of 1.25×10^-5^, 4.96×10^-5^, 5.21×10^-5^, 2.44×10^-4^, 4.08×10^-4^. Scatter plots displaying the significant outcomes of MR effect regarding the impact of cognitive performance (**Figure 3**), self-reported ability (**Figure 4**), educational attainment (**Figure 5**), and completion of highest-level math class (**Figure 6**) on autoimmune disorders, separately. Detailed results are shown in **Table 1** and **Supplementary Table 10**.

**Table 1:** Results of two-sample MR analysis between education and risk of autoimmune diseases.

Exposure	Outcome	N SNPs	Inverse variance weighted					
			beta	se	OR	LCI	UCI	pval
cognitive performance	Autoimmune	271	-0.172682878	0.028304843	0.841404	0.795996596	0.889402503	1.05523E-09
	Psoriasis	271	-0.373941102	0.069055281	0.688017	0.600922411	0.787735621	6.12552E-08
	Rheumatoid arthritis	271	-0.284183799	0.062031243	0.752628	0.666466745	0.849928915	4.62092E-06
	Hypothyroidism	270	-0.182584505	0.041299132	0.833114	0.768333954	0.903356321	9.82337E-06
	Ulcerative colitis	242	-0.281009777	0.080378319	0.755021	0.644970124	0.883849684	0.000472131
educational attainment	Psoriasis	428	-0.494513057	0.081186747	0.609868	0.520149463	0.71506128	1.12168E-09
	Autoimmune	427	-0.20212243	0.033402482	0.816995	0.765220467	0.872272359	1.43842E-09
	Rheumatoid arthritis	429	-0.378116951	0.07208655	0.68515	0.594873448	0.789127549	1.56012E-07
	Hypothyroidism	429	-0.226873575	0.049699192	0.797022	0.723044875	0.878566955	4.99649E-06
	Asthma	439	-0.177764568	0.039456558	0.837139	0.774839459	0.904448679	6.62718E-06
	Crohn’s disease	367	-0.476275652	0.109140415	0.621092	0.501480345	0.769233712	1.27776E-05
	Irritable bowel syndrome	430	-0.320118938	0.082255832	0.726063	0.61795455	0.853083789	9.95247E-05
highest-level math class completed	Psoriasis	404	-0.413976226	0.069417995	0.661017	0.576929331	0.757359713	2.47E-09
	Rheumatoid arthritis	403	-0.336537501	0.061843631	0.714239	0.632704989	0.806280175	5.28E-08
	Autoimmune	402	-0.154453283	0.029044706	0.856884	0.809465677	0.907079067	1.05E-07
	Irritable bowel syndrome	404	-0.319238965	0.070095273	0.726702	0.633417383	0.833724532	5.25E-06
	Hypothyroidism	404	-0.159461896	0.040686424	0.852602	0.787251679	0.923378082	8.88E-05
	Primary biliary cholangitis	148	-0.633260865	0.167368488	0.530858	0.382394262	0.736962237	0.000154556
	Crohn’s disease	346	-0.342438696	0.092997844	0.710037	0.591724295	0.852004968	0.00023121
self-reported math ability	Autoimmune	311	-0.140528496	0.032171725	0.868899	0.815800795	0.925453016	1.25E-05
	Crohn’s disease	268	-0.401083587	0.098848452	0.669594	0.551658241	0.812742766	4.96E-05
	Rheumatoid arthritis	313	-0.268734777	0.066417063	0.764346	0.671049602	0.870613335	5.21E-05
	Irritable bowel syndrome	313	-0.281723693	0.076787961	0.754482	0.649061311	0.877025432	0.000243642
	Psoriasis	312	-0.275794282	0.078016505	0.758969	0.651350987	0.884368072	0.000407658

### Effects of education on mediators

Higher cognitive performance is associated with higher personal pretax income and reduced smoking behavior; the ORs with 95% CI were 1.51 (1.47, 1.55), 0.81 (0.76, 0.85), and the P-values were 2.34×10^-185,^ and 5.82×10^-15^, respectively. Every increase of one standard deviation in educational attainment, equivalent to 4.2 years of schooling, has a positive effect on the average total household income before tax and has a protective effect against smoking and BMI. The ORs with 95% CI for each trait were 1.90 (1.85, 1.96), 0.63 (0.60, 0.67), and 0.86 (0.82, 0.91); the values were less than 2.34×10^-185^, 3.18×10^-58^, and 8.57×10^-8^, respectively. Highest-level math class completed can also increase average total household income before tax and reduce exposure of smoking as well as BM; the ORs with 95% CI for each mediator were 1.64 (1.59, 1.68), 0.70 (0.66, 0.73), and 0.91 (0.87, 0.96). P-values were 6.65×10^-266^, 1.14×10^-49^, and 1.75×10^-4^, respectively. Higher self-reported math ability is related to higher average total household income before tax and decreased smoking behavior. The ORs with 95% CI for these two mediators were 1.43 (1.38, 1.48) and 0.80 (0.75, 0.85), and the P-values were 2.90×10^-95^ and 1.13×10^-13^. There is no significant genetic predisposition of these four education-related exposures to alcohol use (drinks per week).

### Effects of income, smoking, and BMI on autoimmune diseases

Higher average total household income before tax has a protective effect against three traits of autoimmune disorders, including psoriasis, autoimmune diseases, and hypothyroidism; the 95% confidence interval dominance ratios were 0.55 (0.41, 0.74), 0.78 (0.68, 0.89), and 0.77 (0.64, 0.92) with the P-values of 5.73×10^-5^, 2.92×10^-4^, and 5.71×10^-3^, respectively. Smoking can raise the incidence of asthma and RA while reducing PSC, the 95% CI dominance ORs were 1.13 (1.03, 1.24), 1.22 (1.05, 1.43), and 0.63 (0.39, 0.99), and P-values were 7.45×10^-3^, 1.13×10^-2^, and 4.82×10^-2^, respectively; higher BMI increases the risk of autoimmune diseases, psoriasis, asthma, hypothyroidism, multiple sclerosis, RA, asthma and allergy and PBC. The ORs with 95% CI for the above traits were 1.18 (1.13, 1.24), 1.49 (1.33, 1.67), 1.20 (1.14, 1.27), 1.24 (1.15, 1.33), 1.36 (1.20, 1.55), 1.27 (1.15, 1.41), 1.29 (1.09, 1.52), and 1.30 (1.09, 1.54), respectively. The P-values of these autoimmune disorders were 5.89×10^-13^, 2.75×10^-12^, 2.74×10^-11^, 2.43×10^-9^, 2.91×10^-6^, 4.09×10^-6^, 2.60×10^-3^, and 3.40×10^-3^, respectively.

### Mediation of income, smoking, and BMI

As for the genetic predisposition of self-reported math ability on autoimmune disorders, average total household income before tax mediated 78% (95% CI: 20% to 135%) on psoriasis, 63% (95% CI: 18% to 107%) on autoimmune diseases, and 67% (95% CI: 2% to 131%) on hypothyroidism; smoking mediated 164% (95% CI: -516% to 844%) on asthma, 17% (95% CI: 1% to 33%) on RA, and 53% (95% CI: -53% to 160%) on primary sclerosing cholangitis.

Regarding the cause-effect of cognitive performance and autoimmune disorders, it was found that the average total household income before tax acted as a mediator, accounting for 66% (95% CI: 26% to 106%) of the effect, 58% (95% CI: 21% to 96%) on autoimmune diseases, and 60% (95% CI: 10% to 110%) on hypothyroidism; smoking mediated 38% (95% CI: -6% to 81%) on asthma, and 15% (95% CI: 1% to 29%) on RA, and 84% (95% CI: -94% to 235%) on primary sclerosing cholangitis.

In regards to autoimmune disorders and educational attainment, income was found to have a mediating effect of 78% (95% CI: 32% to 124%), specifically on psoriasis, 78% (95% CI: 29% to 128%) on autoimmune diseases, and 75% (95% CI: 13% to 138%) on hypothyroidism while smoking mediated 33% (95% CI: 4% to 61%) on asthma, 24% (95% CI: 3% to 46%) on RA, 47% (95% CI: -13% to 107%) on primary sclerosing cholangitis. BMI mediated 12% (95% CI: 5% to 19%) on autoimmune diseases, 12% (95% CI: 5% to 18%) on psoriasis, 15% (95% CI: 5% to 24%) on asthma, 14% (95% CI: 5% to 22%) on hypothyroidism, 46% (95% CI: -53% to 145%) on multiple sclerosis, 9% (95% CI: 3% to 15%) on RA, 27% (95% CI: -24% to 78%) on asthma and allergy, and 12% (95% CI: -3% to 27%) on primary biliary cholangitis.

Completing the highest-level math class also genetically decreases the risks of autoimmune disorders. Specifically, there is a 71% mediation effect (with a 95% confidence interval of 29% to 113%) of the average total household income before the tax on psoriasis, 78% (95% CI: 27% to 130%) on autoimmune diseases, 82% (95% CI: 11% to 153%) on hypothyroidism. In comparison, smoking mediated 44% (95% CI: 1% to 87%) on asthma, 22% (95% CI: 3% to 40%) on RA, and 38% (95% CI: -9% to 85%) on PSC. The proportion of the indirect effect of BMI on autoimmune disorders was 9% (95% CI: 3% to 15%) on psoriasis, 10% (95% CI: 3% to 17%) on autoimmune diseases, 16% (95% CI: 2% to 31%) on asthma, 12% (95% CI: 2% to 22%) on hypothyroidism, 25% (95% CI: -17% to 67%) on multiple sclerosis, 7% (95% CI: 2% to 12%) on RA, 17% (95% CI: -10% to 44%) on asthma and allergy, 4% (95% CI: 0% to 8%) on PBC. However, it is important to note that not all exposures and outcomes have a significant causal relationship. Therefore, the results of some mediation analyses may not be credible, including the genetic links of self-reported math ability on asthma and PSC; cognitive performance on PSC; and educational attainment on asthma and allergy and PBC. **Table 2** and **Supplementary Table 13** provide comprehensive results.

**Table 2:** Causal effect of education on mediators and of mediators on autoimmune diseases in two-step Mendelian randomization analyses.

Exposure	Mediator	Effect of exposures on mediators				Outcomes	Effect of mediators on outcomes				Proportion of mediary effect			
		OR	LCI	UCI	P		OR	LCI	UCI	P	Proportion	SE	LCI	UCI
cognitive performance	income	1.50769	1.466478	1.550061	2.34E-185	Psoriasis	0.549813	0.410833	0.735808	5.73E-05	0.656785	0.204617	0.255736	1.057835
		1.50769	1.466478	1.550061	2.34E-185	Autoimmune	0.78188	0.68441	0.893232	0.000292	0.585029	0.188915	0.214756	0.955302
		1.50769	1.466478	1.550061	2.34E-185	Hypothyroidism	0.767283	0.635874	0.925849	0.005712	0.595681	0.255003	0.095874	1.095487
educational attainment		1.905603	1.848091	1.964905	0.00E+00	Psoriasis	0.549813	0.410833	0.735808	5.73E-05	0.779967	0.233094	0.323103	1.236831
		1.905603	1.848091	1.964905	0.00E+00	Autoimmune	0.78188	0.68441	0.893232	0.000292	0.784945	0.253283	0.288512	1.281379
		1.905603	1.848091	1.964905	0.00E+00	Hypothyroidism	0.767283	0.635874	0.925849	0.005712	0.752872	0.31896	0.127711	1.378033
highest-level math class completed		1.635315	1.590684	1.681199	6.65E-266	Psoriasis	0.549813	0.410833	0.735808	5.73E-05	0.710681	0.214047	0.291149	1.130213
		1.635315	1.590684	1.681199	6.65E-266	Autoimmune	0.78188	0.68441	0.893232	0.000292	0.783525	0.262694	0.268645	1.298405
		1.635315	1.590684	1.681199	6.65E-266	Hypothyroidism	0.767283	0.635874	0.925849	0.005712	0.817041	0.362489	0.106563	1.527519
self-reported math ability		1.430427	1.382775	1.479722	2.90E-95	Psoriasis	0.549813	0.410833	0.735808	5.73E-05	0.776418	0.294756	0.198697	1.354139
		1.430427	1.382775	1.479722	2.90E-95	Autoimmune	0.78188	0.68441	0.893232	0.000292	0.626781	0.226825	0.182204	1.071359
		1.430427	1.382775	1.479722	2.90E-95	Hypothyroidism	0.767283	0.635874	0.925849	0.005712	0.667055	0.329806	0.020635	1.313476
cognitive performance	smoking	0.807208	0.764956	0.851795	5.82E-15	Asthma	1.13379	1.034161	1.243018	0.007455	0.375113	0.221763	-0.05954	0.809769
		0.807208	0.764956	0.851795	5.82E-15	Rheumatoid arthritis	1.223039	1.046519	1.429333	0.011349	0.151738	0.071181	0.012223	0.291252
		0.807208	0.764956	0.851795	5.82E-15	Primary sclerosing cholangitis	0.626455	0.39393	0.996232	0.048161	0.706859	0.840457	-0.94044	2.354155
educational attainment		0.630071	0.595589	0.666549	3.18E-58	Asthma	1.13379	1.034161	1.243018	0.007455	0.326285	0.143267	0.045481	0.607088
		0.630071	0.595589	0.666549	3.18E-58	Rheumatoid arthritis	1.223039	1.046519	1.429333	0.011349	0.245963	0.108954	0.032413	0.459514
		0.630071	0.595589	0.666549	3.18E-58	Primary sclerosing cholangitis	0.626455	0.39393	0.996232	0.048161	0.471151	0.304755	-0.12617	1.068472
highest-level math class completed		0.695063	0.66241	0.729325	1.14E-49	Asthma	1.13379	1.034161	1.243018	0.007455	0.439509	0.220147	0.008021	0.870998
		0.695063	0.66241	0.729325	1.14E-49	Rheumatoid arthritis	1.223039	1.046519	1.429333	0.011349	0.217621	0.095935	0.029588	0.405653
		0.695063	0.66241	0.729325	1.14E-49	Primary sclerosing cholangitis	0.626455	0.39393	0.996232	0.048161	0.382288	0.240743	-0.08957	0.854144
self-reported math ability		0.797476	0.75123	0.846569	1.13E-13	Asthma	1.13379	1.034161	1.243018	0.007455	1.635822	3.469853	-5.16509	8.436733
		0.797476	0.75123	0.846569	1.13E-13	Rheumatoid arthritis	1.223039	1.046519	1.429333	0.011349	0.169548	0.082232	0.008373	0.330724
		0.797476	0.75123	0.846569	1.13E-13	Primary sclerosing cholangitis	0.626455	0.39393	0.996232	0.048161	0.533246	0.544619	-0.53421	1.600699
educational attainment	BMI	0.865585	0.821038	0.912549	8.57E-08	Autoimmune	1.185114	1.13159	1.24117	5.89E-13	0.121294	0.034619	0.053441	0.189147
		0.865585	0.821038	0.912549	8.57E-08	Psoriasis	1.493734	1.334771	1.671628	2.75E-12	0.117134	0.033603	0.051273	0.182995
		0.865585	0.821038	0.912549	8.57E-08	Asthma	1.201392	1.138239	1.26805	2.74E-11	0.148991	0.048665	0.053607	0.244376
		0.865585	0.821038	0.912549	8.57E-08	Hypothyroidism	1.23817	1.154254	1.328188	2.43E-09	0.135926	0.045277	0.047183	0.224669
		0.865585	0.821038	0.912549	8.57E-08	Multiple sclerosis	1.362429	1.196811	1.550965	2.91E-06	0.46253	0.506158	-0.52954	1.4546
		0.865585	0.821038	0.912549	8.57E-08	Rheumatoid arthritis	1.274386	1.149473	1.412873	4.09E-06	0.092563	0.031843	0.030151	0.154975
		0.865585	0.821038	0.912549	8.57E-08	Asthma and allergy	1.286871	1.092069	1.516421	0.002598	0.273891	0.260531	-0.23675	0.784532
		0.865585	0.821038	0.912549	8.57E-08	Primary biliary cholangitis	1.295683	1.089482	1.540911	0.0034	0.118392	0.078001	-0.03449	0.271273
highest-level math class completed		0.91056	0.867076	0.956224	0.0001748	Autoimmune	1.185114	1.13159	1.24117	5.89E-13	0.103029	0.036519	0.031452	0.174606
		0.91056	0.867076	0.956224	0.0001748	Psoriasis	1.493734	1.334771	1.671628	2.75E-12	0.090822	0.031407	0.029264	0.15238
		0.91056	0.867076	0.956224	0.0001748	Asthma	1.201392	1.138239	1.26805	2.74E-11	0.165425	0.074021	0.020345	0.310505
		0.91056	0.867076	0.956224	0.0001748	Hypothyroidism	1.23817	1.154254	1.328188	2.43E-09	0.125527	0.050864	0.025833	0.22522
		0.91056	0.867076	0.956224	0.0001748	Multiple sclerosis	1.362429	1.196811	1.550965	2.91E-06	0.249999	0.216378	-0.1741	0.674099
		0.91056	0.867076	0.956224	0.0001748	Rheumatoid arthritis	1.274386	1.149473	1.412873	4.09E-06	0.067505	0.026309	0.01594	0.11907
		0.91056	0.867076	0.956224	0.0001748	Asthma and allergy	1.286871	1.092069	1.516421	0.002598	0.169121	0.138208	-0.10177	0.440008
		0.91056	0.867076	0.956224	0.0001748	Primary biliary cholangitis	1.295683	1.089482	1.540911	0.0034	0.038327	0.019445	0.000214	0.076439

### TWAS and GO biological process enrichment analyses

Using the TWAS method, we identified the commonly differentially expressed genes in education and autoimmune diseases. Afterward, we investigated the biological processes enrichment of these differentially expressed genes on the Gene Ontology database. A variety of biological processes were found to be enriched in the genetic predisposition of autoimmune diseases and education, providing MR studies with a potential biological and functional basis for transcriptomics. For the connection of cognitive performance on UC, biological processes including response to arsenic-containing substance (*p* = 7.39×10^-4^), cellular response to arsenic-containing substance (*p* = 2.96×10^-4^) were highly enriched; as for the association cognitive performance of RA, biological processes including positive regulation of protein targeting to membrane (*p* = 1.24×10^-3^) and regulation of protein targeting to membrane (*p* = 1.82×10^-3^) were significantly enriched; positive regulation of cold-induced thermogenesis (*p* = 3.67×10^-4^) fatty acid oxidation (*p* = 4.62×10^-4^), and lipid oxidation (*p* = 5.42×10^-4^) may participate in the development of psoriasis; protein polymerization (*p* = 6.27×10^-5^) and actin polymerization or depolymerization (*p* = 1.35×10^-4^) were highly enriched in the genetic association of cognitive performance on hypothyrodism; meiosis-related cell cycle signaling pathways including meiosis I (*p* = 4.57×10^-4^), meiosis I cell cycle process (*p* = 5.42×10^-4^), and chromosome organization involved in meiotic cell cycle (*p* = 1.35×10^-3^)may participate in the occurrence of autoimmune disorders related to the cognitive performance. The detailed results of GO: BP enrichment analyses are in **Supplementary Tables 14–18**. Visualization of enrichment analysis results is shown in **Supplementary Figures 9–13**.

### Sensitivity analyses

MR sensitivity methods include MR-Egger intercept testing, leave-one-out analyses, and Cochran’s Q test, which can ensure the results’ stability. The MR-Egger intercept (**Supplementary Table 11**) testing showed no horizontal pleiotropy in the significant results of education-related factors on the autoimmune disorders. The funnel plot (**Supplementary Figures 1–4**) also proved the absence of pleiotropy. Cochran’s Q test (**Supplementary Table 12**) showed heterogeneity. However, it does not affect the results of IVW, and our conclusion is still reliable. Leave-one-out analyses (**Supplementary Figures 5–8**) showed that when a single SNP was gradually removed for analyses, error lines were all on the left side of zero, indicating that a single SNP does not significantly affect the overall outcome.

## Discussion

As far as we know, this is the first extensive analysis using MR that thoroughly investigates the genetic predisposition of cognitive performance, educational attainment, highest-level math class completion, and self-reported math ability on 22 traits of autoimmune disorders (AA, SLE RA, psoriasis, hypothyroidism, hyperthyroidism, ILD, AIHA, SS, scleroderma, AS, autoimmune diseases, asthma, ALS, CD, CeD, IBS, PBC, PSC, MS, T1D, and UC), and the potential mediating effect of BMI, income, smoking and alcohol use. The GWAS dataset used in this study is sizeable and can potentially mitigate the weak instrument bias in the MR study. In the present MR analysis, we determined that the genetic predisposition of higher levels of education-related factors led to lower relative odds (OR) of several autoimmune diseases, including psoriasis, hypothyroidism, asthma, RA, CD, UC, and IBS to different degrees. The influence of education-related factors on autoimmune diseases seems partially explained by smoking, income, and BMI, accounting for 10% to 80% of the effects.

A growing body of research has determined that social determinants of health (SDH), especially education-related factors, have an increasingly close relationship to a variety of diseases, including neoplastic diseases (45), cardiovascular diseases (46, 47), autoimmune diseases, etc. Several cross-section and cohort studies have demonstrated that higher education-related factors can protect the occurrence and progression of autoimmune diseases. For instance, a study conducted in Denmark that analyzed 61,153 children discovered that those who had atopic dermatitis (AD) had a higher likelihood of not achieving lower secondary education and upper secondary education (48). E Portaccio et al. (49) investigated 115 adult-onset MS and 111 pediatric-onset MS patients and found that cognitive performance and educational attainment have a protective effect on socio-professional outcomes, and lower education levels also increase the inflammatory activity in MS (50).

However, autoimmune diseases are an extremely large spectrum of diseases, and research on the genetic predisposition of educational factors on many autoimmune diseases is still lacking. A significant advantage of this study is that MR can evaluate both causal and mediating effects. This not only minimizes the confounding errors that arise in observational experiments but also eliminates the ethical and economic issues associated with randomized controlled trials. In our MR study, we found that a higher level of education can genetically decrease the incidence of various autoimmune disorders; we then investigated the potential mediators of the genetic predisposition between education and autoimmune disorders.

To obtain possible mediating effects, we utilized a two-step MR analysis for potential mediators and their proportion mediating effect of the total genetic cause-effect of the education-related factors on autoimmune diseases. According to a multivariable two-sample MR analysis conducted by Davis NM et al. (51), obtaining a higher level of education has been found to have a positive impact on income and alcohol consumption while showing negative effects on smoking, BMI, and sedentary behavior. Therefore, we chose BMI, average total household income before tax, smoking, and alcohol use as mediators. The results of the two-step MR analysis suggested that income, smoking, and BMI mediated different cause-effect of exposures and outcomes to different degrees, which was similar to the former MR study conducted by Ferguson LD (52), which demonstrated that the central adiposity is related to psoriasis and RA. The result of the mediating effect of BMI and smoking on educational attainment on RA also agreed with Zhao SS et al. (53). In contrast, the mediating effect of alcohol use was insignificant, which corresponded with what Bae SC et al. (54) and Wei J et al. (55) did before. Smoking has been demonstrated to increase the risks of MS (56), RA (57), and other autoimmune disorders (58). Higher BMI is also related to higher incidence and poorer treatment response of RA, SLE, IBD, and psoriasis (59). The biological mechanism of mediating the effect of BMI may relate to the pro-inflammatory processes of adipose tissue, which can decrease Bregs and Tregs, promote the activation of Th17 and Th1 cells (60), and generate dysregulated intestinal flora metabolites (61). Smoking can cause oxidative stress and produce ROS and other free radicals, generating autoreactive pro-inflammatory T cells, autoantibodies, and pro-inflammatory cytokines (58).

We then conducted TWAS and GO: BP enrichment analyses to explore the biological mechanisms that may explain the genetic link between education and autoimmune diseases. Because of the integrity of the GWAS database, we only studied the relationship of cognitive performance on UC, RA, psoriasis, hypothyroidism, and autoimmune disease. According to our enrichment analyses, biological processes, including response to arsenic-containing substance and protein transcription, may participate in the occurrence of UC. Trivalent arsenic [As(III)] has recently been found to be an immunomodulatory agent (62), and the organic arsenic derivative acetarsol has been studied for mesalazine-refractory UC and showed some curative effects (63). The exposure to As can influence the induction and modulation of regulatory T cells, thereby decreasing immune surveillance and increasing autoimmune disease risk (64). Biological processes, such as protein localization, play a role in the genetic association of cognitive performance on RA, including regulating protein targeting to the membrane. The distinct expressions of the regulators of protein trafficking and the membrane adapter protein are essential indicators of metabolically reprogrammed T cells. Naive CD4 T cells can turn into pro-inflammatory helper T cells through protein targeting process, which contributes to the development of RA (65). Fatty acid oxidation and lipid oxidation participate in the development of psoriasis. The oxidative stress in psoriasis increases the production of lipid mediators, particularly eicosanoids. These are vital for the differentiation of Th1 and Th17 cells and the modulation of cellular immunity in psoriasis patients (66). Abnormal lipid metabolism can lead to ferroptosis, and inhibiting keratinocyte ferroptosis can suppress psoriatic inflammation by reducing cytokine production (67). Actin polymerization or depolymerization may partially explain the relationship between cognitive performance and hypothyroidism. An animal study found that defects in AFAP1L2 can affect cellular polarity and cytoskeletal structure, resulting in epithelial function disorders like congenital hypothyroidism (68). Targeting this process in the future could be an effective method for regulating immune cells and controlling autoimmune diseases.

This study has several limitations. First, although some cohort studies suggest that autoimmune diseases may impede academic development in patients (69–72), we were unable to conduct a reverse MR study due to the incomplete summary-level GWAS statistics of the exposure. Second, compared to other observational designs, MR is less prone to confounding as genetic variants are predetermined; the population stratification can still be a potential confounder at the sample level. This occurs when there is a correlation resulting from sub-populations that have varying distributions of genetic variants and exposure/outcome (73, 74). For example, the summary level of GWAS data for this MR analysis was limited to those of Europeans, and the incidence of autoimmune diseases varies among different races, ethnicities, and regions (75). Additionally, we identified smoking, alcohol consumption, BMI, and income as potential factors that could help explain the link between education and autoimmune diseases based on previous research. However, it’s important to note that other factors, such as external environment, self-induced stress, and dietary habits, may also play a role but were not taken into consideration in this study. Last, MR may not accurately capture changes in exposures over time, and a single BMI measurement may not provide a complete picture of BMI throughout one’s life (76).

In conclusion, our two-sample and two-step MR showed a protective effect of higher levels of education-related factors (including cognitive performance, educational attainment, highest-level math class completed, and self-reported math ability) on particular autoimmune diseases (including psoriasis, hypothyroidism, asthma, RA, CD, UC, and IBS); encouraging population-level interventions to decrease smoking, manage excess weight, and promote income equality could effectively mitigate the increased risks. However, the impact of education on autoimmune diseases is still largely uncertain, but it’s certain that enhancing access to education and controlling modifiable factors, including BMI and smoking, are vital measures for lower risk of autoimmune diseases and better health outcomes. Moreover, it’s imperative to conduct more studies on environmental risk factors that can be altered to reduce the elevated morbidity of autoimmune diseases caused by a lower level of education.

## Data availability statement

The original contributions presented in the study are included in the article/**Supplementary Material**. Further inquiries can be directed to the corresponding author.

## Author contributions

Writing -Original Draft, Methodology, Validation, Visualization: YJL and JZ. Data Collection and Validation: JW, ML, and WL. Conceptualization, Methodology, Supervision, Project Administration, and Funding Acquisition: YZL. All authors contributed to the article and approved the submitted version.
