# Supplementary material for: Large-scale genome-wide association study to identify causal relationships and potential mediators between education and autoimmune diseases
Source: Front Immunol. 2023 Dec 7;14:1249017. doi: 10.3389/fimmu.2023.1249017 (PMC10749315; doi:10.3389/fimmu.2023.1249017)
Supplement: Supplementary file 1 [file DataSheet_1.docx]

Supplementary Information


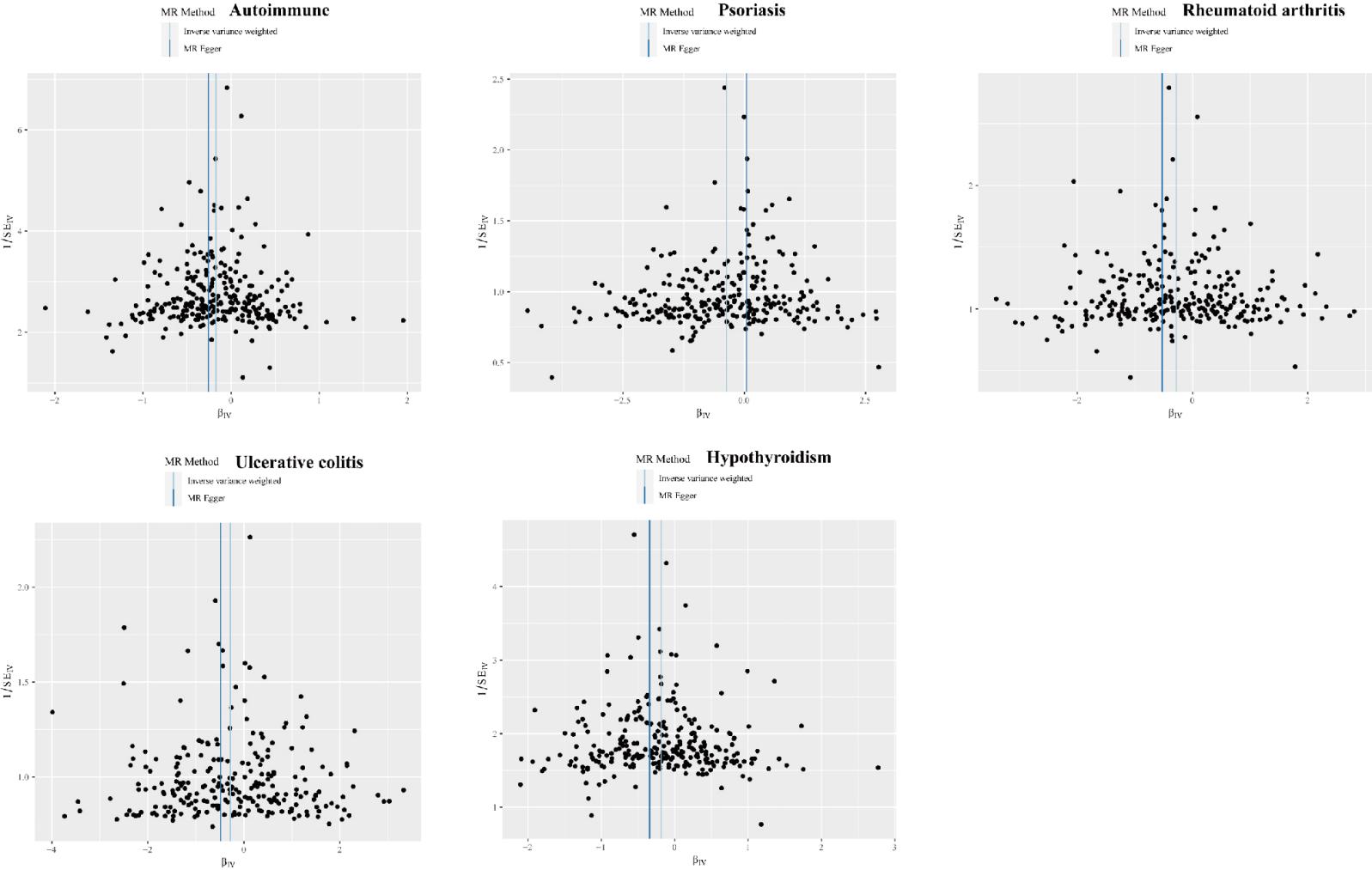


**Figure S1.** Funnel plot from genetically predicted cognitive performance on autoimmune diseases.


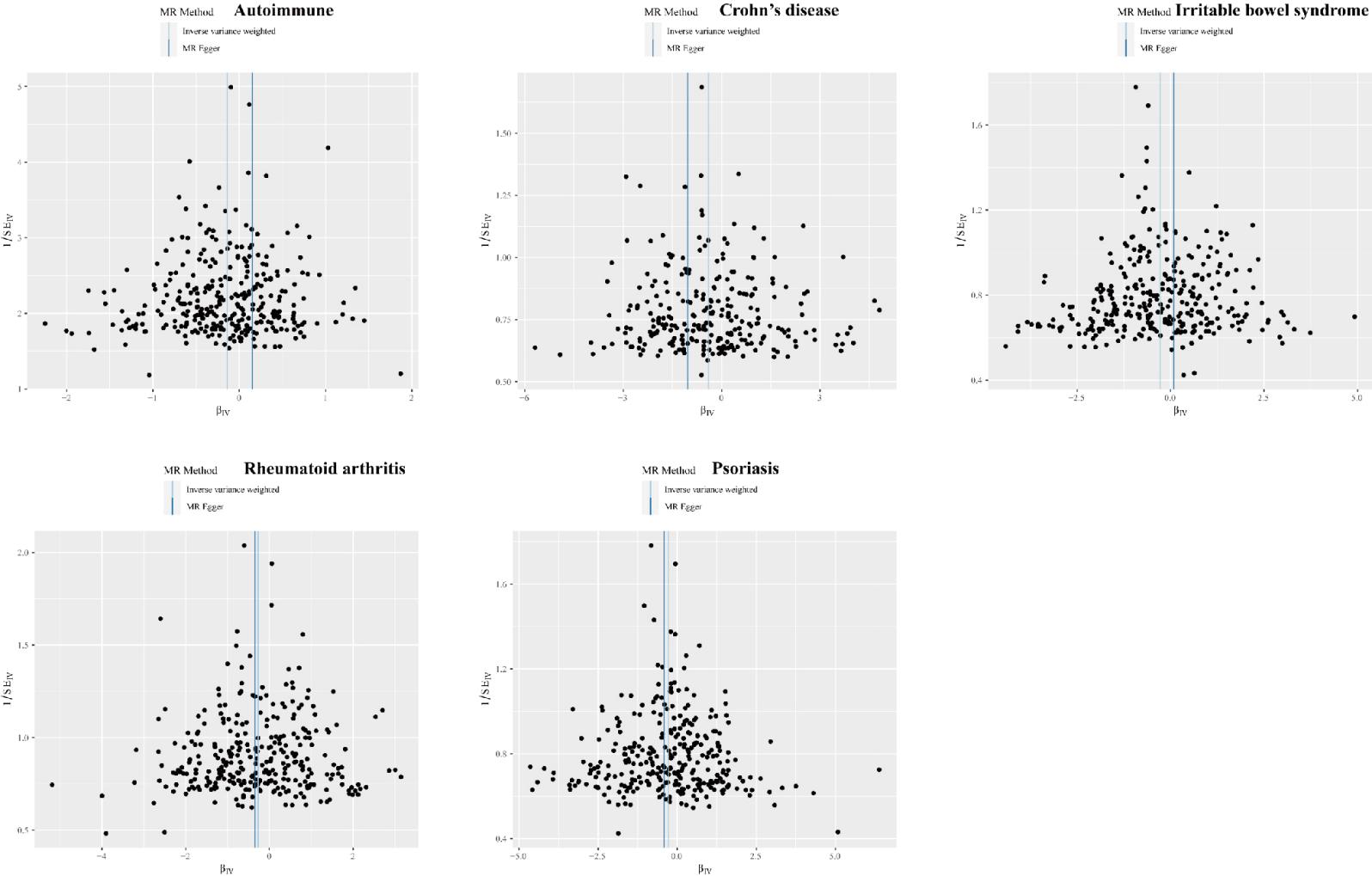


**Figure S2.** Funnel plot from genetically predicted self-reported math ability on autoimmune diseases.


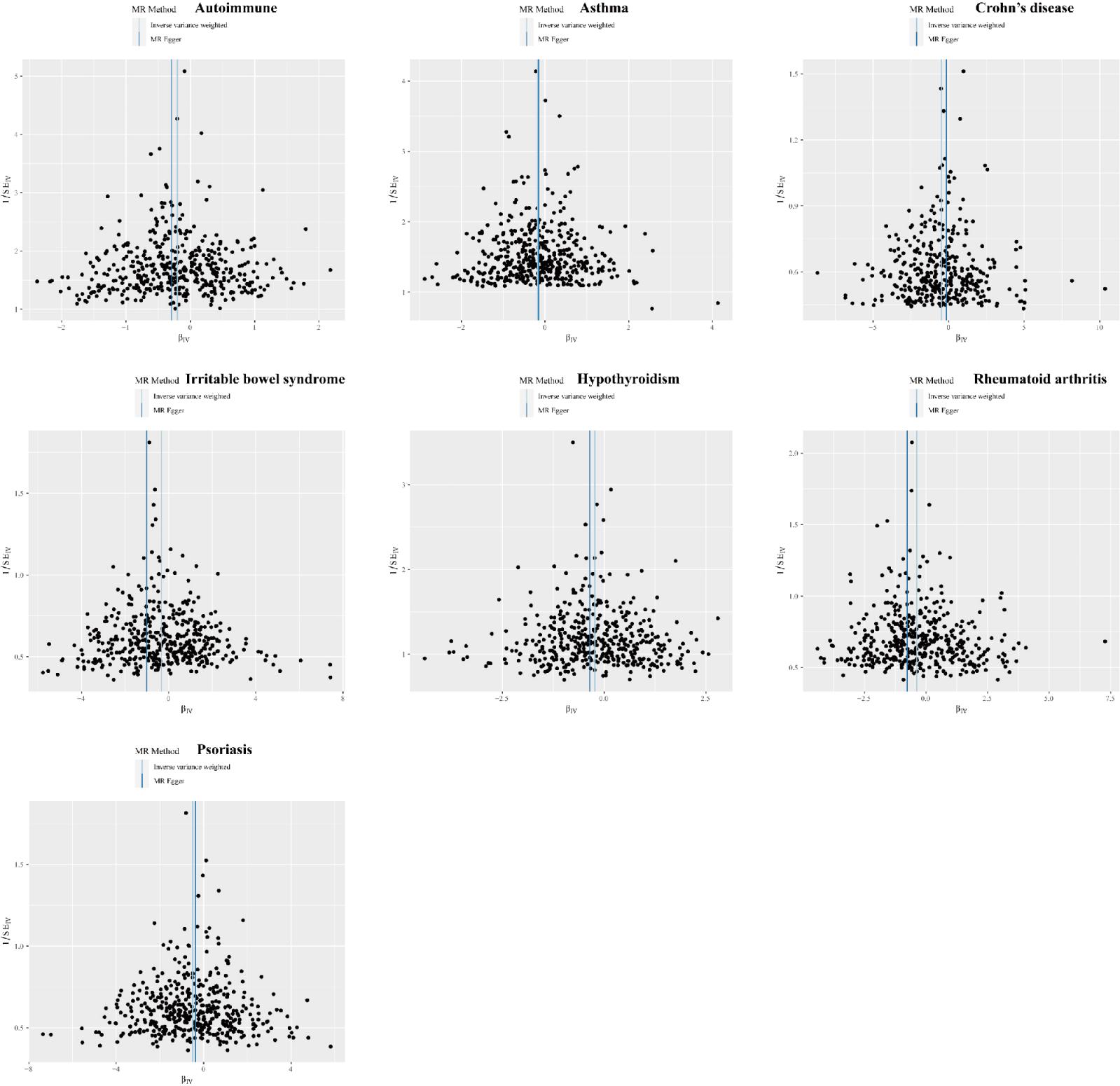


**Figure S3.** Funnel plot from genetically predicted educational attainment on autoimmune diseases.


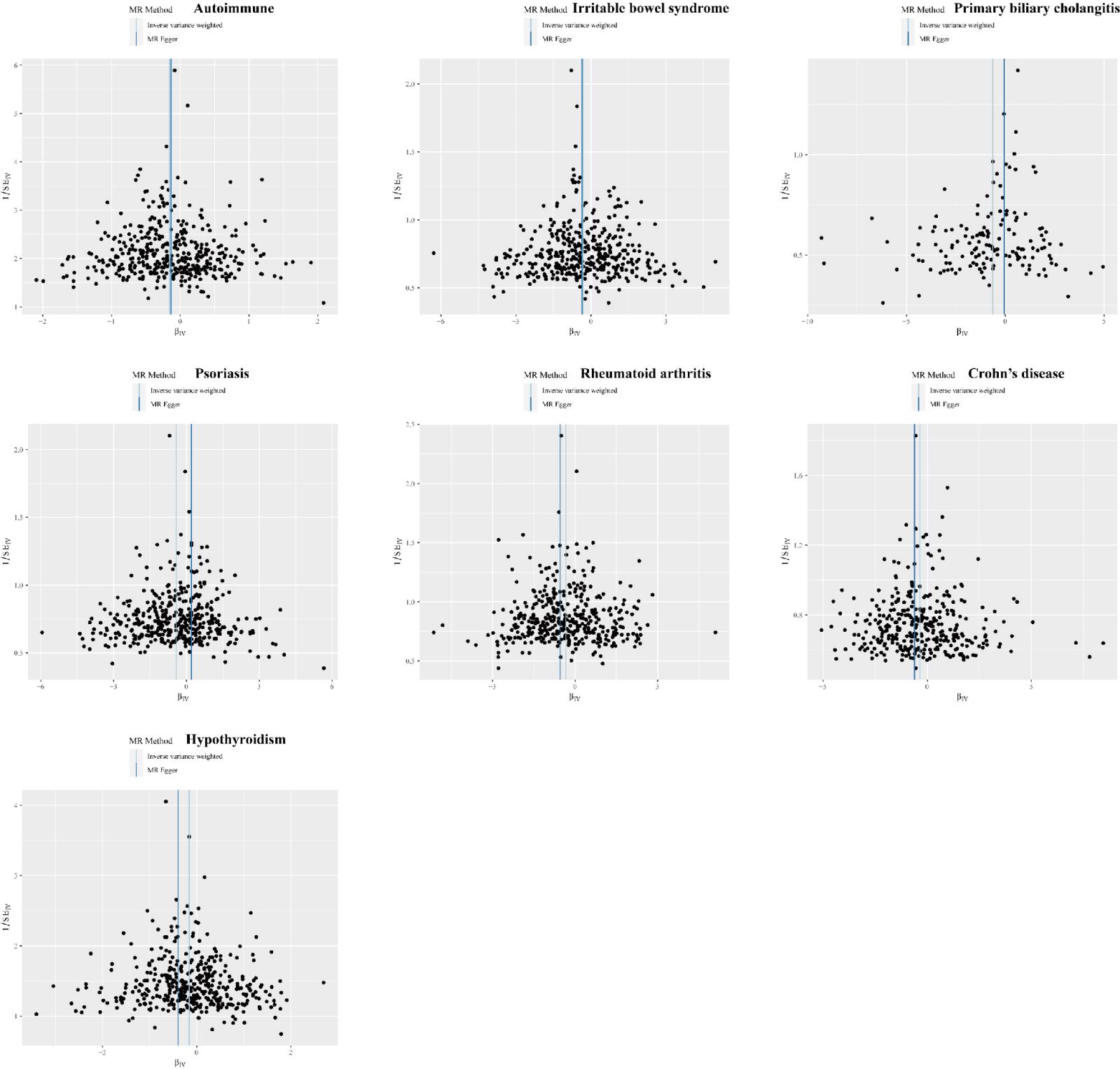


**Figure S4.** Funnel plot from genetically predicted highest-level math class completed on autoimmune diseases.


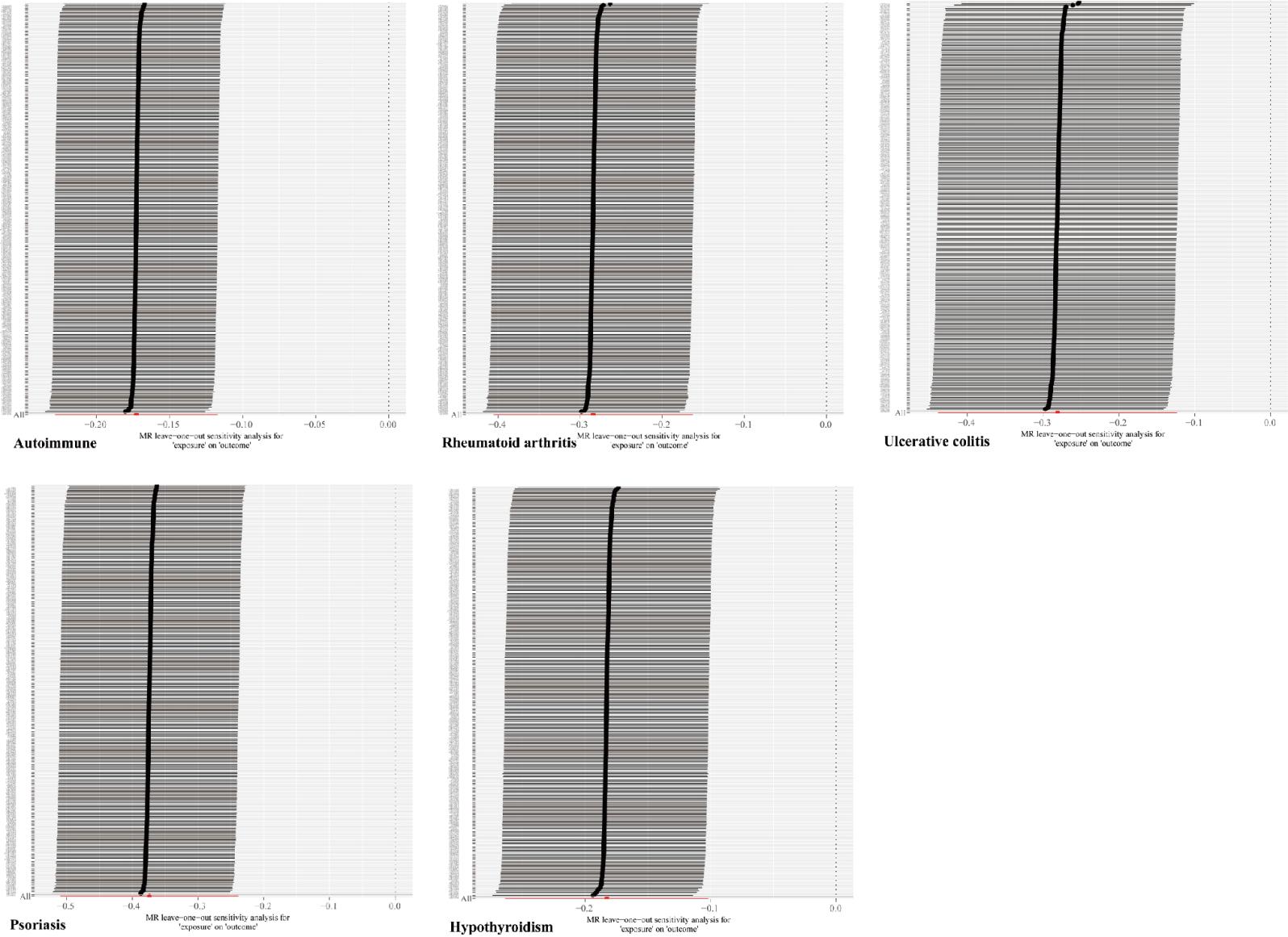


**Figure S5.** The leave-one-out plot from genetically predicted cognitive performance on autoimmune diseases.


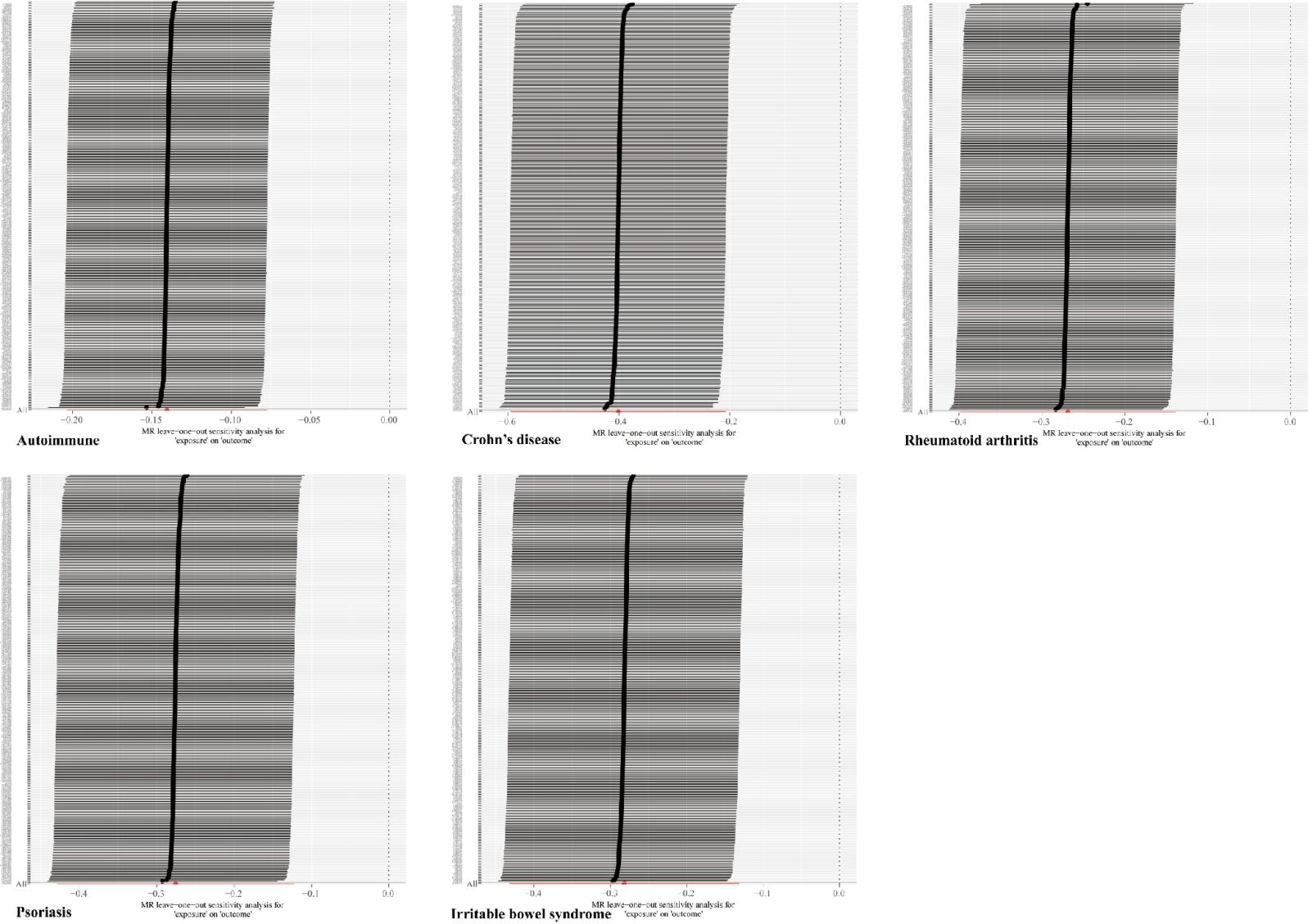


**Figure S6.** The leave-one-out plot from genetically predicted self-reported math ability on autoimmune diseases.


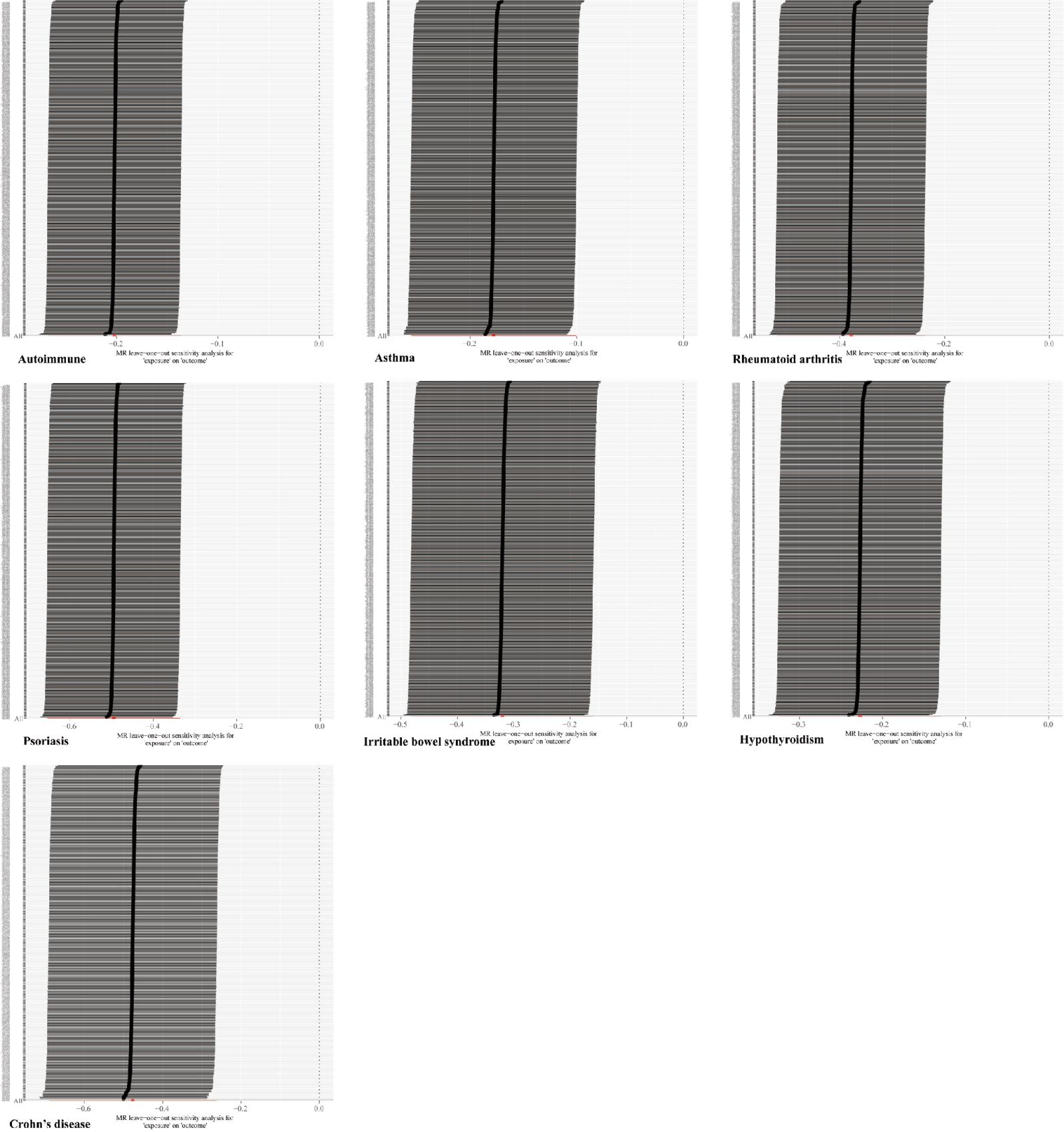


**Figure S7.** The leave-one-out plot from genetically predicted educational attainment on autoimmune diseases.


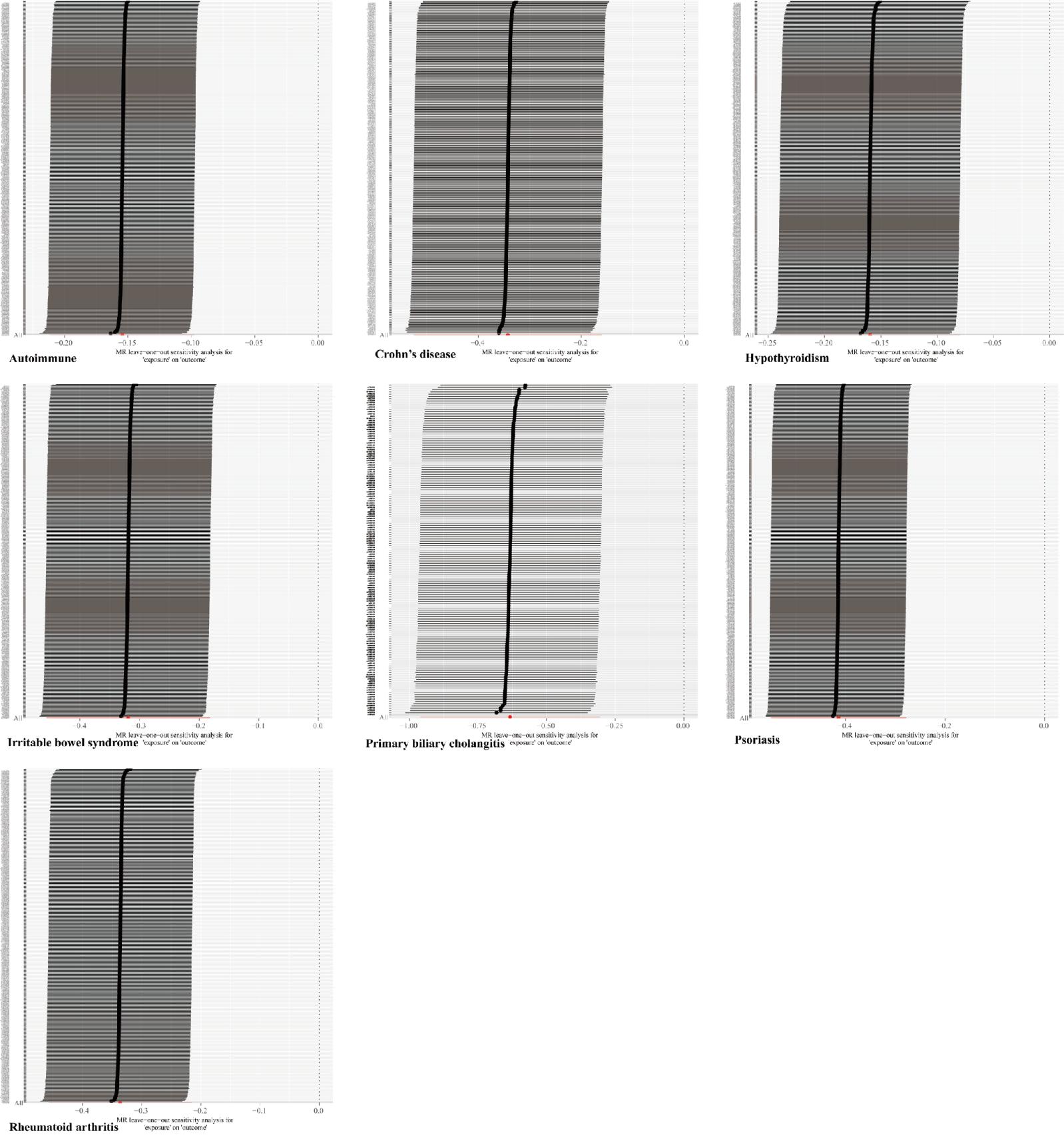


**Figure S8.** The leave-one-out plot from genetically predicted highest-level math class completed on autoimmune diseases.


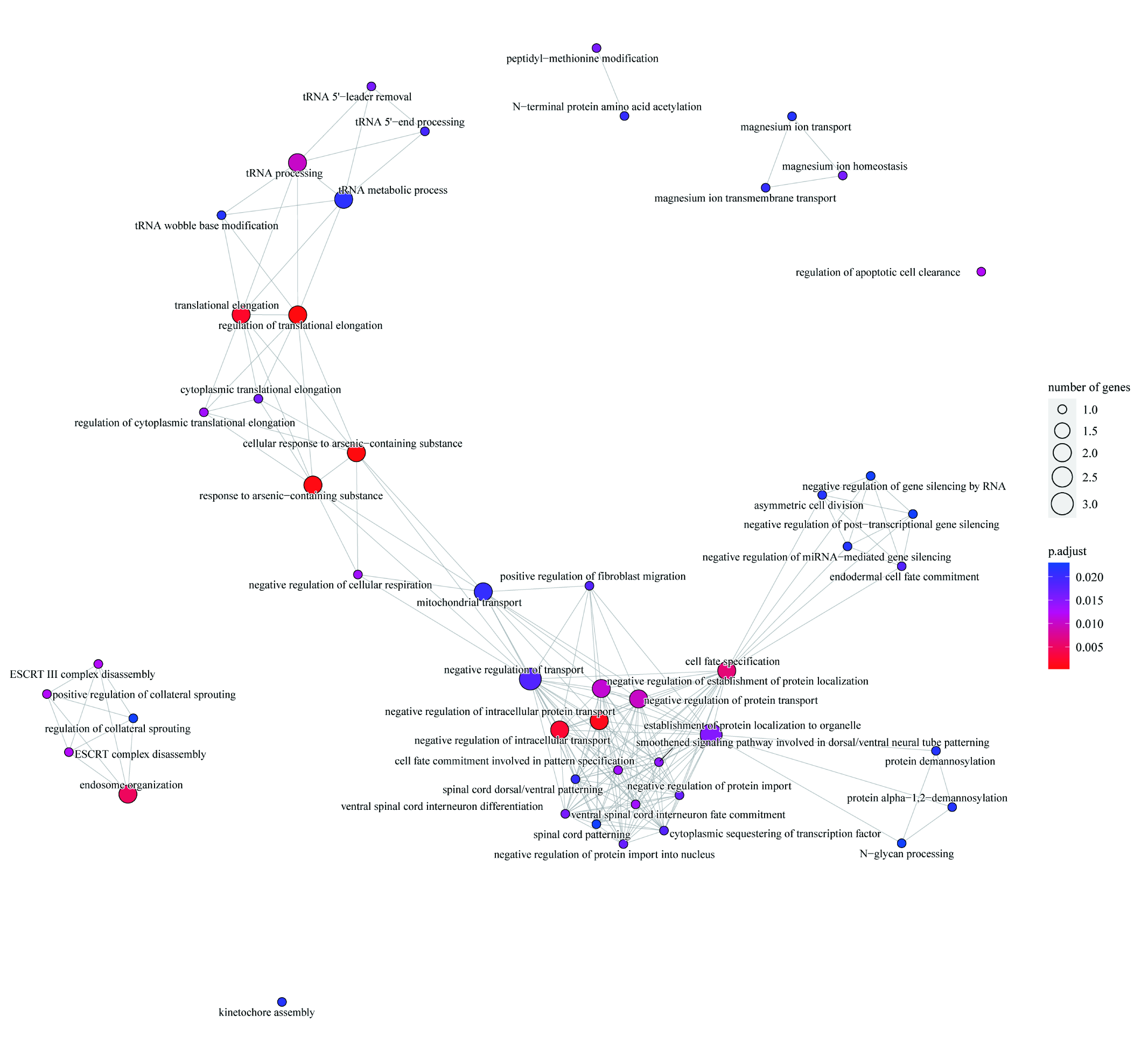


**Figure S9.** Enrichment pathways analysis associated with ulcerative colitis and cognitive performance.


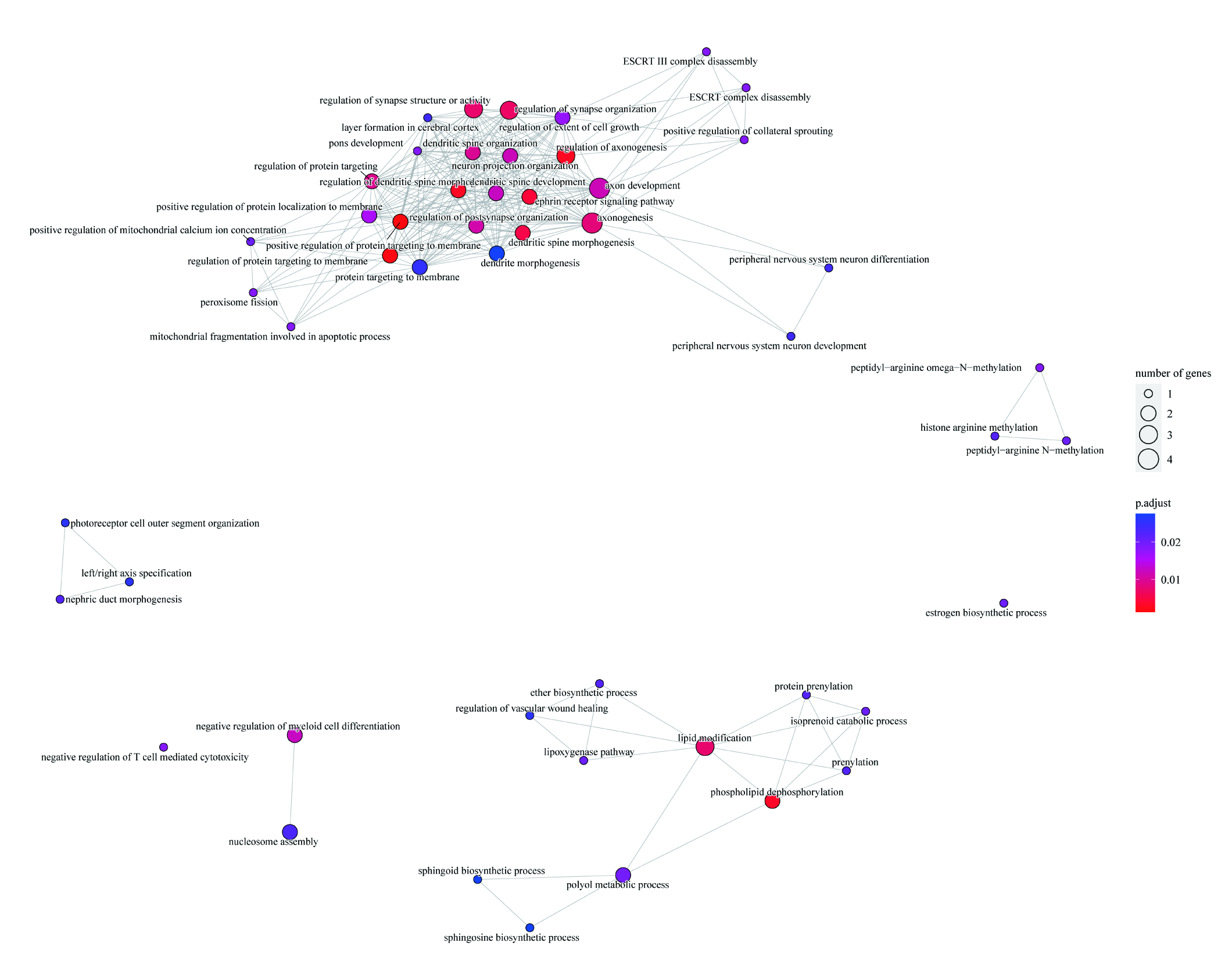


**Figure S10.** Enrichment pathways analysis associated with rheumatoid arthritis and cognitive performance.


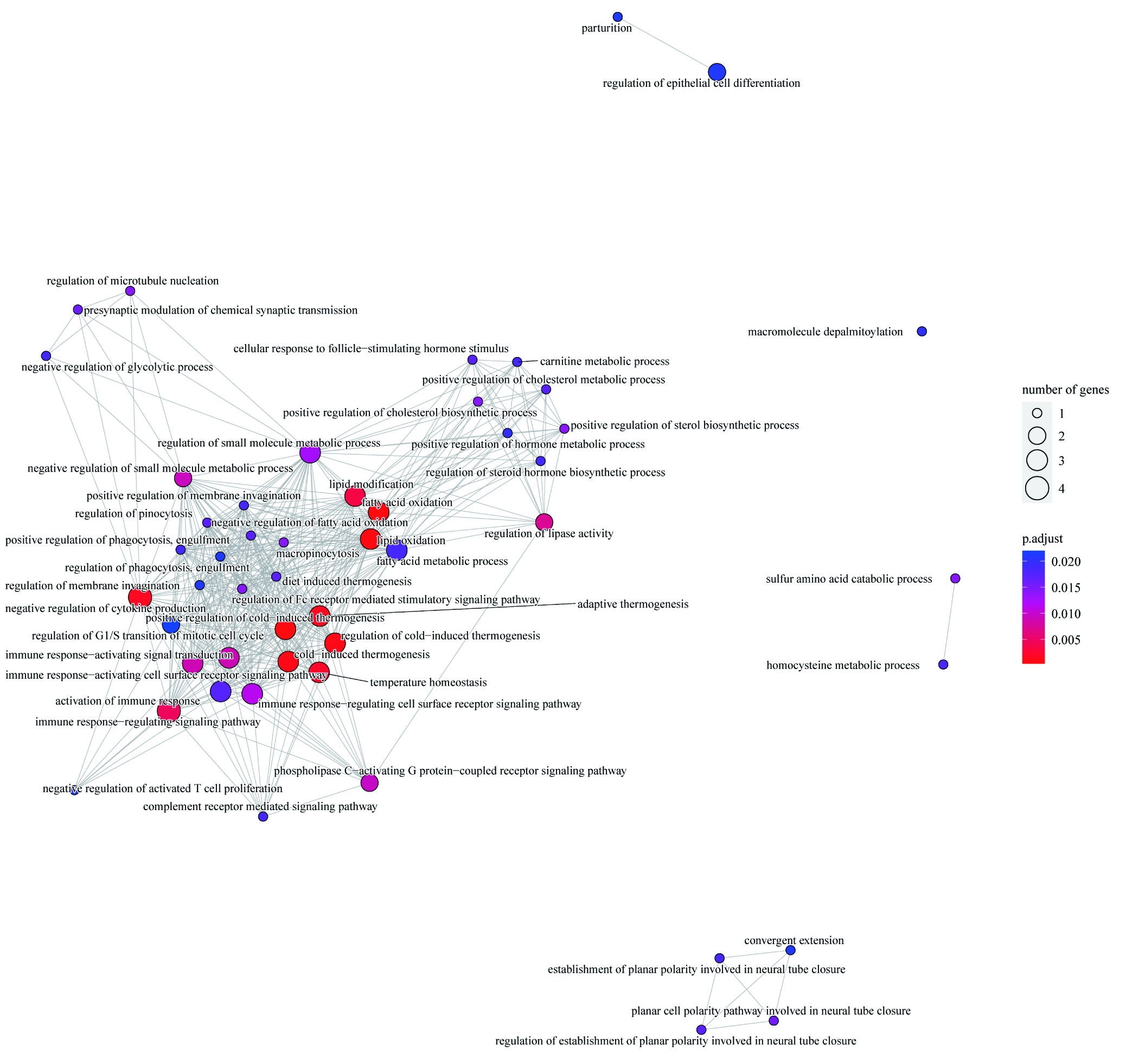


**Figure S11.** Enrichment pathways analysis associated with psoriasis and cognitive performance.


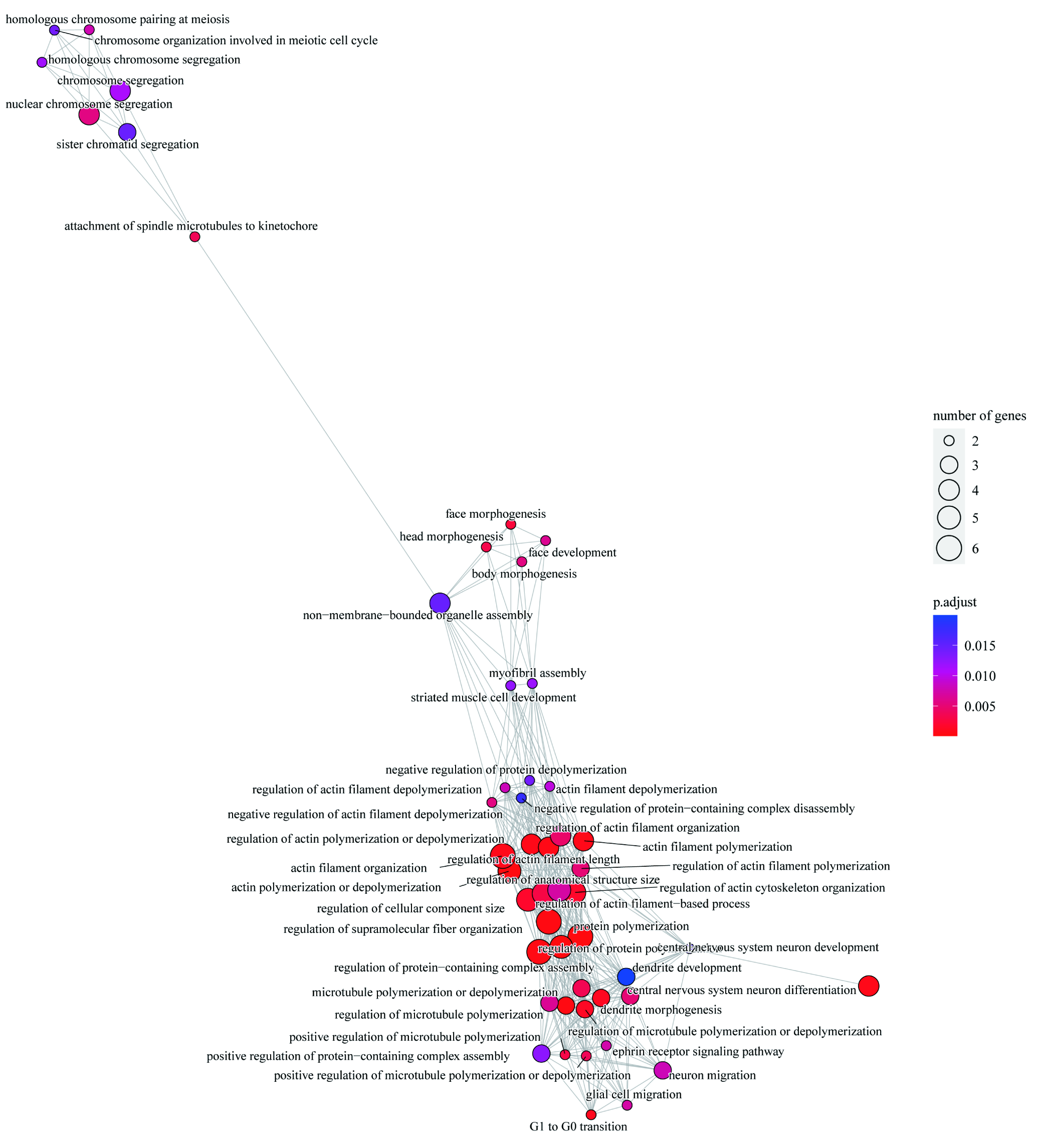


**Figure S12.** Enrichment pathways analysis associated with hypothyroidism and cognitive performance.


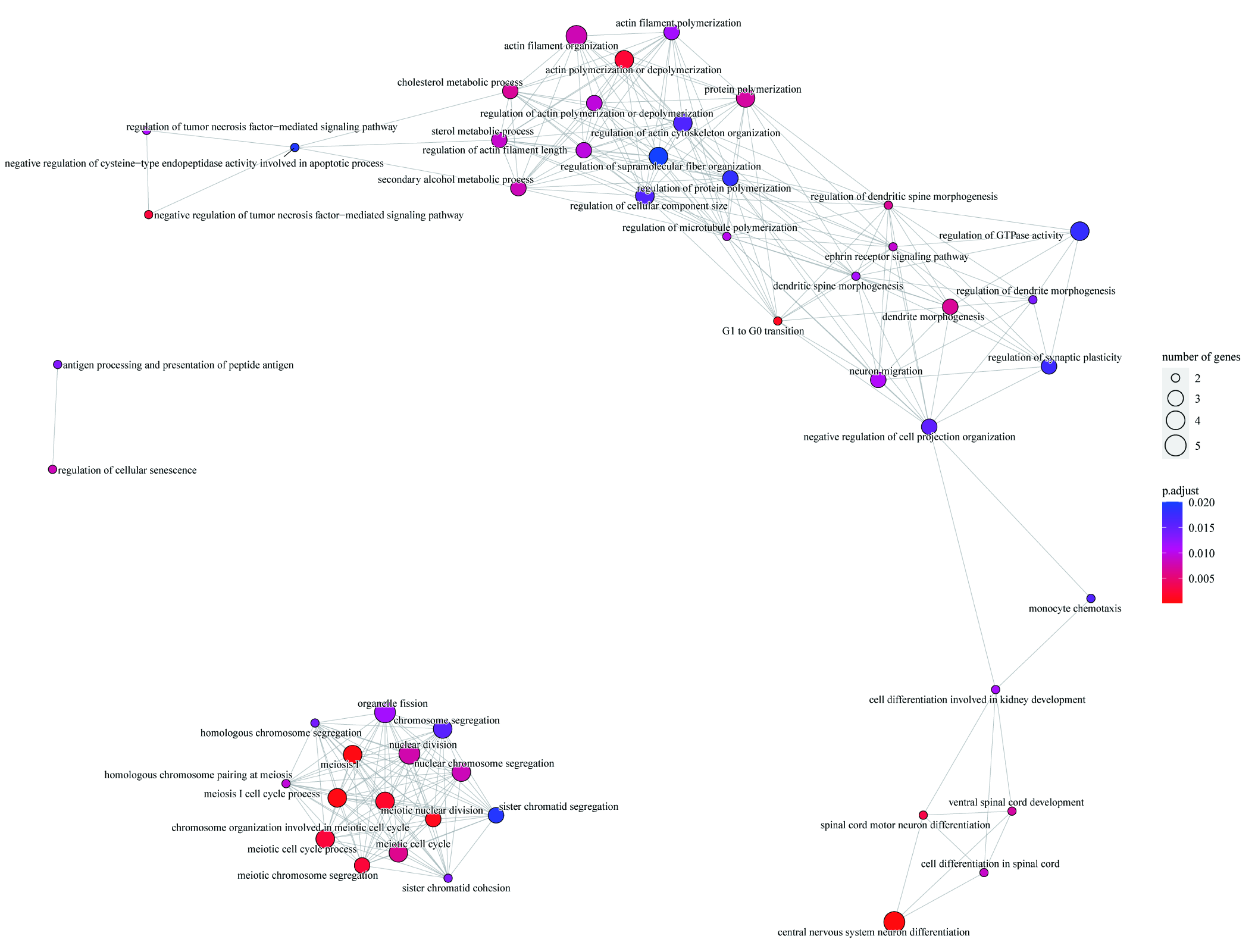


**Figure S13.** Enrichment pathways analysis associated with autoimmune disease and cognitive performance.
